# Activation and Characterization of Lanthomicins A–C by Promoter Engineering in *Streptomyces chattanoogensis* L10

**DOI:** 10.3389/fmicb.2022.902990

**Published:** 2022-05-10

**Authors:** Xiao-Fang Liu, Jun-Xiao Wang, Xin-Ai Chen, Yu Liu, Yong-Quan Li

**Affiliations:** ^1^First Affiliated Hospital and Institute of Pharmaceutical Biotechnology, Zhejiang University School of Medicine, Hangzhou, China; ^2^Zhejiang Provincial Key Laboratory for Microbiol Biochemistry and Metabolic Engineering, Hangzhou, China; ^3^College of Life Science, Zhejiang University, Hangzhou, China

**Keywords:** cryptic gene cluster, lanthomicin, antiproliferative activity, CRISPR-Cpf1, *Streptomyces chattanoogensis* L10

## Abstract

The emergence of drug resistance highlights the importance of new drug discovery. Microbial secondary metabolites encoded in biosynthetic gene clusters (BGCs) are a prolific source of drugs, whereas most of these BGCs are cryptic. Thus, taking strategies to activate these cryptic BGCs is of great importance for potential drug discovery. In this work, three novel pentangular polyphenols lanthomicin A–C were identified by activating a cryptic aromatic polyketide BGC through promoter engineering combined with optimization of fermentation conditions. We further confirmed the involvement of lanthomicin (*ltm*) BGC in biosynthesis by CRISPR-Cpf1-assisted gene editing. Based on functional analysis of homologous genes, a putative biosynthetic pathway was proposed for the three lanthomicins. Particularly, lanthomicin A showed antiproliferative activity with IC_50_ 0.17 μM for lung cancer cell line A-549. The discovery of lanthomicins brings new members to the pentangular polyphenol subclade of aromatic polyketide and demonstrates the potential of *Streptomyces* as a source for drug discovery.

## Introduction

*Streptomyces* genus has been one of the richest sources of bioactive natural products since the early 1950s (Baral et al., 2018). Indeed, a vast majority of compounds originating from this genus are clinically used in medicine, such as daptomycin (Vilhena and Bettencourt, 2012), doxorubicin (Arcamone et al., 1969), and FK506 (Rath, 2013). However, the drug discovery reached a bottleneck based on the classic bioactivity-guided approaches, while the drug resistance became a major threat to modern healthcare (Strachan and Davies, 2017). One promising approach to address this question comes with the development of genome sequencing technology. Genome mining of *Streptomyces* has revealed that they have far greater potential to produce specialized metabolites than have ever been estimated, based on the plethora of BGCs identified (Kalkreuter et al., 2020). Since it is by now well-established that most natural product BGCs are silent or poorly expressed under standard laboratory conditions that the cognate products do not accumulate to detectable levels (Nett et al., 2009). Therefore, the key question remaining to answer is how to access these hidden potentials.

In the light of huge sequence data in the genomic era, several main strategies have been developed in an attempt to harness this tremendous chemical diversity. These include promoter engineering (Li et al., 2017), diversification of culture conditions (Zhang et al., 2014), ribosome engineering (Wang et al., 2008; Thong et al., 2016), manipulation of regulators (Chen et al., 2017), and others. Among them, promoter engineering has been proved as a powerful and efficient method to activate or improve the biosynthesis of interest products (Li et al., 2015, 2017). *Streptomyces chattanoogensis* L10 is an industrial producing strain of natamycin, which is synthesized by type I polyketide synthases and widely used as an antifungal agent in both drug therapy and the food industry (Aparicio et al., 2016; Meena et al., 2021). Researchers have also activated the cryptic BGCs of chattamycins (Zhou et al., 2015) and anthrachamycin (Li et al., 2019) in *S. chattanoogensis* L10 by overexpressing regulatory gene and ribosome engineering, respectively. Both of them fell into the angucycline clade of aromatic polyketides. It seems that *S. chattanoogensis* L10 is inclined to produce polyketides, suggesting the potential to be developed as chassis for expressing exogenous BGCs (Bu et al., 2019).

In this study, we identified and activated a cryptic BGC of lanthomicin (*ltm*) by promoter engineering combined with optimization of fermentation conditions. Three newly synthesized lanthomicins were purified and their chemical structures were elucidated. We found that the lanthomicins represent new members of the pentangular polyphenols family. Cytotoxicity assay revealed that lanthomicin A possesses potential antiproliferative activity.

## Materials and Methods

### Strains and Media

The strain *S. chattanoogensis* L10 was used as a starting strain for genome mining. *Escherichia coli* DH5α was used as a general plasmid-cloning host. *E. coli* ET12567/pUZ8002 (Kieser et al., 2000) was used to introduce plasmids into *Streptomyces* by interspecies conjugation. *S. chattanoogensis* L10 and recombinant strains were grown at 30°C on YMG solid medium (yeast extract 0.4%, malt extract 1%, glucose 0.4%, agar 2%, and CaCO_3_ 0.2%) for genetic screen and spore preparation (Supplementary Table 1). A total of 3% tryptic soy broth was used for vegetative growth of *Streptomyces* for genomic DNA extraction. MS solid medium (mannitol 2%, soybean flour 2%, and agar 2%) was used for exoconjugants growth. YEME liquid medium (yeast extract 0.3%, malt extract 0.3%, tryptone 0.5%, and glucose 4%) was used to produce lanthomicins. *E. coli* strains were cultured in a Luria-Bertani medium. When required, media were supplemented with antibiotics at the following final concentrations: chloramphenicol (25 μg/ml), nalidixic acid (25 μg/ml), and kanamycin (50 μg/ml). Spectinomycin was used in a final concentration of 50 μg/ml for *E. coli*, while 200 μg/ml for *Streptomyces*.

### General DNA Manipulation

Polymerase chain reaction (PCR) was performed with KOD FX DNA polymerase (TOYOBO, Japan) for high-fidelity cloning and with rTaq DNA polymerase (Takara) for colony screening according to the instructions of the manufacturers. Gene fragments were purified by a gel extraction kit (Magen Biotech Co.) and plasmids were recovered from a plasmid extraction kit (Shanghai Generay Biotech Co.). DNA concentration was measured by a Nanodrop Lite spectrophotometer (Thermo Scientific Co.). Genomic DNA isolation of *S. chattanoogensis* L10 and its derivatives was performed as described (Kieser et al., 2000). *Streptomyces* colony PCR was also performed for rapid genotype validation: mycelium from single clones was scraped with a sterilized toothpick and ultrasonication in FTPS (10 mM Tris-HCl; 0.5 mM EDTA; and 0.1 mM KCl) solution, 1 μl of the solution was then used as a template for reactions.

### Plasmid Construction

Primers used in this study were listed in Supplementary Table 3. Genomic DNA isolated from the L10 strain was used as a template for PCR amplification. The fidelity of amplified fragments was confirmed by DNA sequencing. pSET152 and pKC1139 were digested with *Sac*I. The spectinomycin resistance gene was cloned by PCR with a primer pair spec-F/R using plasmid pIJ778 (Gust et al., 2003) as a template and then inserted into the above-digested vectors by seamless cloning, generating pSET152-spec and pKC1139-spec. For the purpose of constructing a universal vector to overexpress genes, pSET152-spec was digested with *Xba*I and *EcoR*V. A 97 bp PCR fragment of the kasO^*^ promoter amplified with primers kasO^*^p-F/R was inserted into pSET152-spec, affording plasmid pSET152-spec-kasO^*^. Detailed procedures for plasmid construction were described in Supplementary Methods. All the plasmids used in this work were listed in Supplementary Table 2.

### High-Performance Liquid Chromatography (HPLC) and Liquid Chromatography-Mass Spectrometry (LC-MS) Analysis

Fermentation broth of *S. chattanoogensis* L10 and recombinant strains from YEME were mixed with two volumes of methanol. After centrifugation for 5 min at 14,000 rpm (Eppendorf Centrifuge 5424R), the supernatant was filtered for high-performance liquid chromatography (HPLC) analysis, which was performed on an Agilent Liquid Chromatograph 1,260 (Agilent, Inc) equipped with a 4.6 × 150 mm Extend-C18 column, with a linear gradient of 5–100% MeCN-H_2_O in 30 min followed by 100% MeCN for 5 min and 5% MeCN for another 5 min in a flow rate of 1 ml/min. LC-MS analysis was performed in an Agilent 1200 HPLC system (Agilent, Santa Clara, CA, USA) and a Termo Finnigan LCQDeca XP Max LC/MS system (Termo Finnigan, Waltham, MA, USA). Agilent Extend-C18 was used as the column, and H_2_O (0.1% formic acid) and acetonitrile were used as the mobile phases A and B with a linear gradient from 5% to 100% (v/v) B over 30 min.

### Cultivation, Extraction, and Isolation of Compounds

To accumulate enough compounds, L10 strains were cultivated in a 15 L fermenter at 30°C in YEME media for 7 days. When the fermentation was stopped, the fermentation broth was first adjusted pH to be lower than 4.0 with 2M hydrochloric acid solution and extracted three times using an equal volume of ethyl acetate. After removing the solvent in vacuo, the extract was fractioned by silica gel chromatography. Then, the fractions containing target compounds were subjected to Sephadex LH-20 column chromatography eluted with pure methanol. Purified extract was further refined by semipreparative liquid chromatography on an Agilent Eclipse XDB-C18 column (5 μm, 9.4 × 250 mm) using acetonitrile and 0.1% HCOOH (v/v) as eluent, yielding 5.4 mg of **1**, 7 mg of **3**, and 11.4 mg of **4**.

### Antiproliferative Assay

The cytotoxicity of lanthomicins A, B, and C was evaluated using the lung cancer cell line A-549, the breast carcinoma cell line MCF-7, the liver cancer cell line HepG2, and the colon carcinoma cell line HCT-116. HCT-116 cells were grown in McCoy's 5A Modified Medium (Gibco) supplemented with 10% fetal bovine serum (FBS). A-549, MCF-7, and HepG2 cells were grown in a DMEM medium (Gibco) supplemented with 10% FBS. Cells in log phase growth were harvested by trypsinization. Trypsinized cells were seeded onto 96-well plates (20,000 cells/ml) and incubated overnight at 37°C in the presence of 5% CO_2_. Cells were transferred to a fresh medium supplemented with lanthomicins A–C (in dimethyl sulfoxide (DMSO)) in different final concentrations and incubated for 72 h to detect the cell viability using CCK8-based colorimetric assays. Doxorubicin was used as a positive control and DMSO was used as a negative control. Cell viability was recorded based on the percent stain present in each well relative to no drug DMSO control wells.

## Results

### Characterization of the Lanthomicin Gene Cluster From *S. chattanoogensis* L10

**Figure 1:**
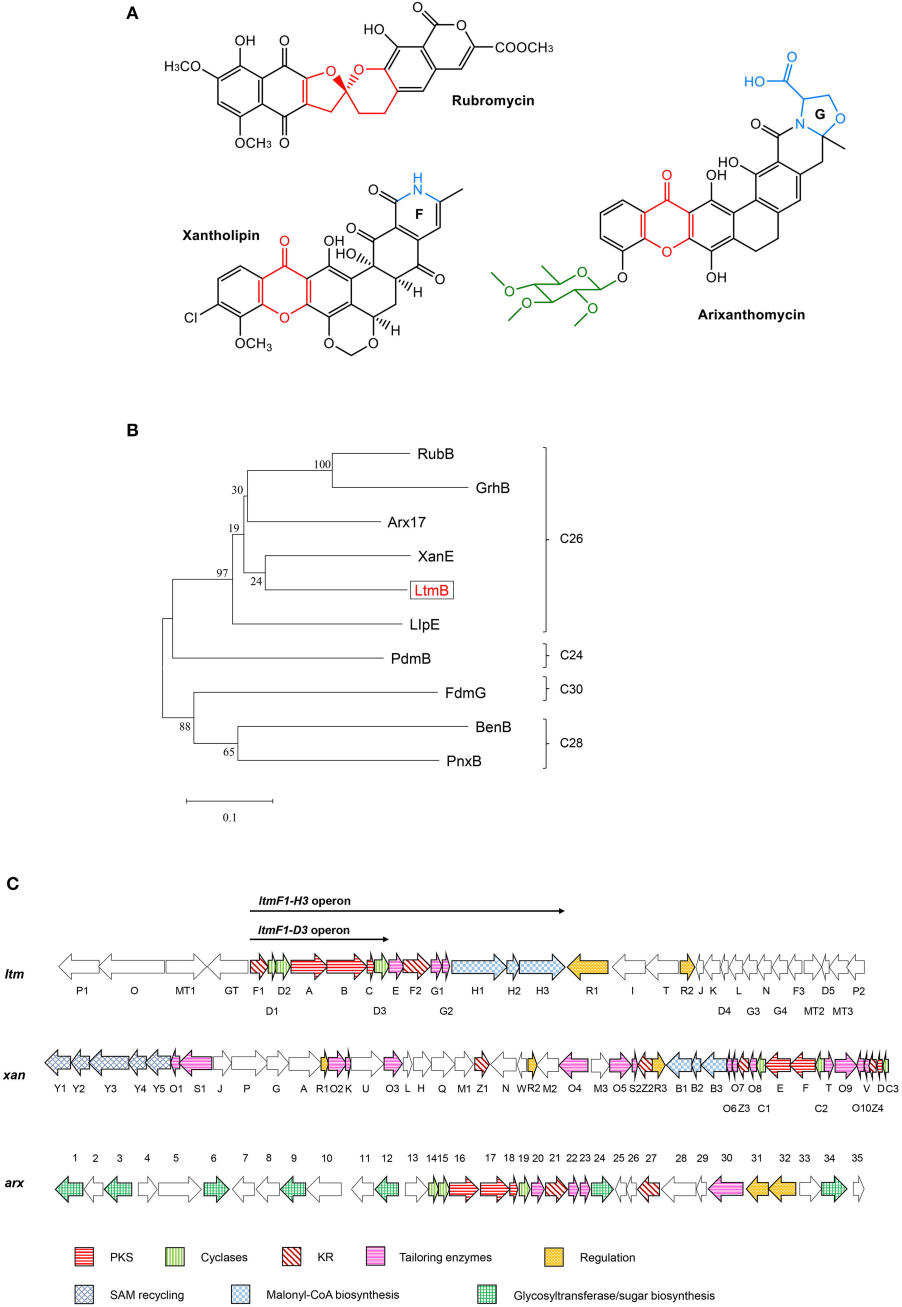
Comparative analysis of the lanthomicin biosynthetic gene cluster. **(A)** Examples of compounds belong to the pentangular polyphenol subfamily. **(B)** The maximum likelihood tree of LtmB with homologous KS_β_ proteins from the well-studied pentangular polyphenol clusters. These KS_β_ units could be divided into four groups according to the carbon chain length of their products. **(C)** *ltm* cluster was indicated by comparison with *xan* and *arx* clusters. The operons for promoter engineering were also indicated in the figure.

To get insight into the BGCs of *S. chattanoogensis* L10, we reanalyzed the completely sequenced genome (CGMCC 2644) using antiSMASH. It was found to possess 37 BGCs, namely, 8 polyketide synthases (PKSs), 2 nonribosomal peptide synthases (NRPSs), and one PKS-NRPS hybrid. A few terpene and lassopeptide BGCs were identified by the presence of corresponding synthase genes (Supplementary Table 4). Among the predicted BGCs, a type II PKS gene cluster (*ltm*, cluster 1) (Figure 1C) was shown to have about 50% homologies to known gene clusters of *xan* and *arx*, which are responsible for the biosynthesis of xantholipin and arixanthomycin, respectively (Zhang et al., 2012; Kang and Brady, 2014). Hence, we supposed the gene cluster *ltm* may be involved in the biosynthesis of pentangular polyphenols which belong to one subclade of the aromatic PKS family. Phylogenetic analysis also found that ketosynthase beta subunit (KS_β_) LtmB from *ltm* gene cluster fell into the same subclade with a 26-carbon skeleton as that of *xan* and *arx* gene clusters (Figure 1B). The core skeleton of this subfamily was synthesized by minimal type II PKS (min-PKS), cyclases, and ketoreductases (KRs) in similar steps. Then the core structure was modified by various tailoring enzymes, which contributed to structural diversity. Examples of compounds from this subclade were listed in Figure 1A and distinctive structures were shown in color.

According to bioinformatic prediction, the *ltm* gene cluster consists of 34 genes (Table 1), including three FAD-dependent monooxygenases, among which LtmG3 showed considerable homology to XanO4 (70% identity) and Arx30 (64% identity). Phylogenetic analysis implied LtmG3 could be classified into an atypical Baeyer–Villiger monooxygenase branch (Supplementary Figure 1). LtmO, which strongly resembled the asparagine synthase homolog XanA (56% identity) and the amidotransferase homolog Arx5 (44% identity), also possessed five conserved amino acid sites for ligand binding and catalyzing (Supplementary Figure 2). Three methyltransferases were there, among them LtmMT2 showed the highest (68%) identity to XanM3 which directed the remethylation of C17 hydroxyl after a cryptic demethylation step catalyzed by XanO4 (Kong et al., 2016). Two cytochrome P450 oxidases LtmP1 and LtmP2 were found in the *ltm* cluster. Three enzymes, LtmD4, LtmD5, and LtmJ, whose exact functions were uncharacterized all distributed among these clusters.

**Table 1:** Deduced functions of genes in the lanthomicin biosynthesis gene cluster and protein homologs annotation.

**Gene product**	**aa**	**Proposed function**	**Most similar protein (acc.number)**	**Identical aa (%)**	**Homologous gene in *xan*, *arx***	**Identity/similarity (%)**
ORF11	62	Transposase	WP_161968789.1	69		
LtmP1	403	Cytochrome P450	WP_137814202.1	83	XanO2	35/52
LtmO	620	Asparagine synthase	WP_137814203.1	85	XanA, Arx5	56/67, 44/58
LtmMT1	328	O-methyltransferase	WP_107082228.1	76	Arx6	39/58
LtmGT	354	Glycosyltransferase	WP_137814140.1	76	XanG, Arx3	55/66, 42/58
LtmF1	208	3-oxoacyl-ACP reductase	WP_137814141.1	86	XanS2, Arx4	53/64, 55/66
LtmD1	111	Cyclase	WP_137814142.1	87	XanC3, Arx14	67/82, 80/92
LtmD2	145	Cyclase	WP_137814143.1	82	XanC2, Arx15	68/79, 70/76
LtmA	422	Beta-ketoacyl synthase alpha	WP_137814144.1	90	XanF, Arx16	80/88, 78/87
LtmB	397	Ketosynthase chain-length factor	WP_137814129.1	87	XanE, Arx17	73/80, 76/83
LtmC	84	Acyl carrier protein	WP_137814128.1	72	XanD, Arx18	40/60, 51/73
LtmD3	152	Cyclase	WP_137814127.1	87	XanC1, Arx19	65/76, 70/82
LtmE	153	Monooxygenase	WP_049718470.1	79	XanO8, Arx20	66/77, 64/73
LtmF2	250	3-oxoacyl-ACP reductase	WP_137814126.1	85	XanZ3, Arx21	65/82, 64/78
LtmG1	108	Monooxygenase	WP_116198857.1	86	XanO7, Arx22	58/74, 67/80
LtmG2	92	Monooxygenase	WP_137814125.1	84	XanO6, Arx23	62/72, 66/76
LtmH1	571	carboxyl transferase alpha	WP_125308160.1	77	XanB3	67/76
LtmH2	174	biotin carboxyl carrier protein	WP_137814199.1	76	XanB2, Arx35	53/60, 49/62
LtmH3	469	biotin carboxylase	WP_137814198.1	88	XanB1	74/83
LtmR1	613	SARP family transcriptional regulator	WP_198535620.1	73		
LtmI	540	FAD-dependent monooxygenase	WP_200827518.1	74	XanO5, Arx10	45/58, 41/57
LtmT	477	MFS transporter	WP_137814146.1	75		
LtmR2	228	TetR family transcriptional regulator	WP_158879437.1	86		
LtmJ	149	Monooxygenase	WP_043782530.1	78	XanO10, Arx25	72/81, 63/78
LtmK	292	Dehydrogenase	WP_137814130.1	79	XanS1, Arx13	53/67, 50/63
LtmD4	128	CurD-like protein	WP_049718476.1	86	XanV, Arx26	76/87, 80/89
LtmL	493	Peptide permease	KNB50326.1	73	XanQ, Arx28	52/68, 49/64
LtmG3	541	FAD-dependent monooxygenase	WP_137814132.1	89	XanO4, Arx30	70/82, 64/77
LtmN	286	Reductase	WP_137814133.1	88	XanZ1, Arx33	57/70, 45/64
LtmG4	421	FAD-dependent monooxygenase	WP_192909516.1	83	XanO4	26/39
LtmF3	245	3-oxoacyl-ACP reductase	WP_137814135.1	88	XanZ4, Arx27	65/77, 67/79
LtmMT2	336	Methyltransferase	WP_137814136.1	86	XanM3	68/80
LtmD5	122	CurD-like protein	WP_137814137.1	83	XanT, Arx29	63/79, 61/75
LtmMT3	351	Methyltransferase	WP_162834049.1	80	XanM3	45/62
LtmP2	467	Cytochrome P450	WP_043782514.1	82	XanO2	39/55
ORF47	191	Transcriptional regulator NovG	GCB88085.1	70		

### Activation of *ltm* by Promoter Knock-In and Variating Fermentation Environment

**Figure 2:**
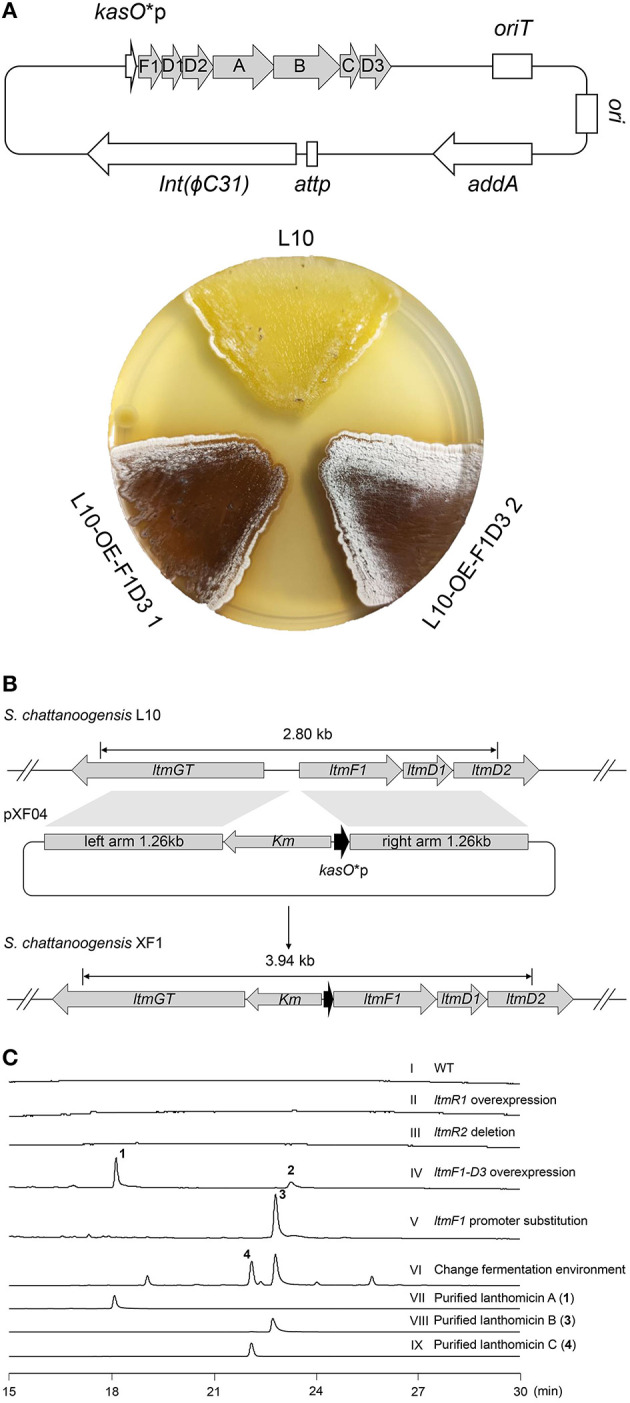
Activation of cryptic lanthomicin gene cluster by promoter knock-in and changing cultivating environment. **(A)** Lanthomicins were produced on a solid YMG plate by overexpression of *ltmF1-D3* cassette. **(B)** Schematic of a promoter replacement strategy that a *Km*-kasO*p cassette was inserted upstream of the *ltmF1* gene and replaced the native promoter by homologous recombination. **(C)** HPLC analysis of the metabolites from different strains (UV at 480 nm): (I) *S. chattanoogensis* L10; (II) *S. chattanoogensis* L10-OE-R1; (III) *S. chattanoogensis* L10-ΔR2; (IV) *S. chattanoogensis* L10-OE-F1D3; (V) *S. chattanoogensis* XF1; (VI) *S. chattanoogensis* XF1 (changing fermentation environment); (VII) isolated lanthomicin A; (VIII) isolated lanthomicin B; and (IX) isolated lanthomicin C.

As Figure 1C shows, two pathway-specific regulatory genes are in the *ltm* cluster, a SARP family regulator-encoding gene *ltmR1* and a TetR family regulator-encoding gene *ltmR2*. Usually, the TetR family regulators play a negative control in antibiotic biosynthesis, while SARP family regulators showed a positive role (Krause et al., 2020). Therefore, we attempted to overexpress the activator gene *ltmR1* under the control of the strong promoter kasO*p and delete the repressor gene *ltmR2* based on homologous recombination. The resulting recombinant strains L10-OE-R1 and L10-ΔR2 were cultivated for analysis of SMs as compared with starter strain L10. However, the metabolite profiles did not show any differential peaks (Figure 2C II and III), indicating that overexpression and/or deletion of the single regulatory gene was not sufficient to activate *ltm* cluster.

To induce the expression of the *ltm* gene cluster, we further try to overexpress the core genes involved in polycyclic skeleton biosynthesis that would bypass the real regulatory pathway. The structural genes (*ltmA-C*), polyketide cyclase genes (*ltmD1-D3*), and a 3-oxoacyl-acyl carrier protein (ACP) reductase gene (*ltmF1*) seemed to be located in a single multicistron (Figure 1C), we cloned this multicistronic cassette (*ltmF1-D3*) into the integrative vector pSET152-spec-kasO^*^, in which the strong promoter kasO^*^p initiated their transcription. The resulting plasmid pXF03 was introduced into L10 to yield L10-OE-F1D3. As expected, overexpression of core gene cassette led to the production of brown pigments both on solid YMG plate and in liquid YEME media, suggesting the activation of gene cluster *ltm* successfully (Figure 2A; Supplementary Figure 3). Metabolites analysis of fermentation broth by HPLC showed that there are two new compounds produced in the overexpressed strain L10-OE-F1D3, lanthomicin A (**1**) and compound **2** (Figure 2C IV).

**Figure 3:**
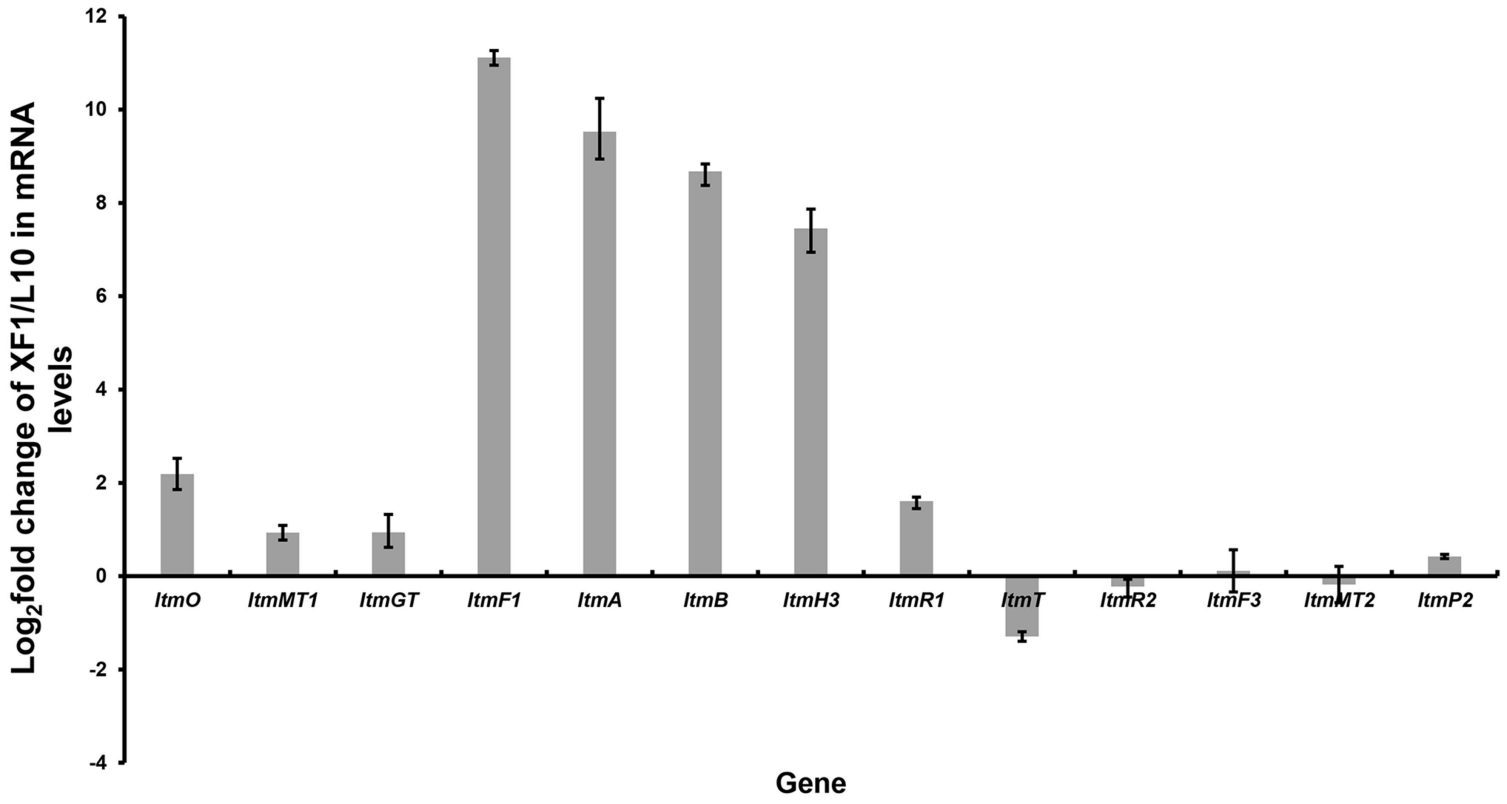
Quantitative real-time PCR experiment. By promoter replacement, gene transcription levels from *ltmF1* to *ltmH3* in *S. chattanoogensis* XF1 were significantly upregulated, while genes distributed at two sides were almost the same transcriptional levels as the control strain L10. Fold change was indicated as log_2_ (XF1/L10).

Inspired by the above results, we found that just downstream of the seven-gene multicistron, three monooxygenase genes (*ltmE, ltmG1-G2*), another one 3-oxoacyl-ACP reductase gene (*ltmF2*), and three genes (*ltmH1-H3*) serving the synthesis of malonyl-CoA extender unit, are arranged in the same direction as the *ltmF1-D3* gene cassette (Figure 1C). We hypothesized that all these genes might be transcribed together to form a bigger transcript. So we replaced the native promoter with the strong promoter kasO^*^p, which was inserted upstream of the *ltmF1* gene by homologous recombination (Figure 2B). Finally, the generated strain *S. chattanoogensis* XF1 was confirmed by PCR (Supplementary Figure 4). The corresponding fermentation result revealed a new compound lanthomicin B (**3**) was synthesized (Figure 2C V). By changing the fermentation environment, we found that fermenting in a 15 L fermentor could produce several new homologs of **3** compared with a flask, and two of them displayed a distinct UV absorption spectrum (Figure 2C VI; Supplementary Figure 5). The difference in absorption spectra might imply structural change and we would like to identify the chemical alteration of lanthomicin C (**4**) with a higher yield. We also conducted the quantitative real-time PCR (qRT-PCR) experiment, the transcriptional levels of genes involved in the activated multicistron were significantly upregulated compared to the control strain L10, while genes distributed at two sides were almost the same transcriptional levels as the control strain (Figure 3).

### Confirmation of Lanthomicins Biosynthesis Gene Cluster *ltm*

**Figure 4:**
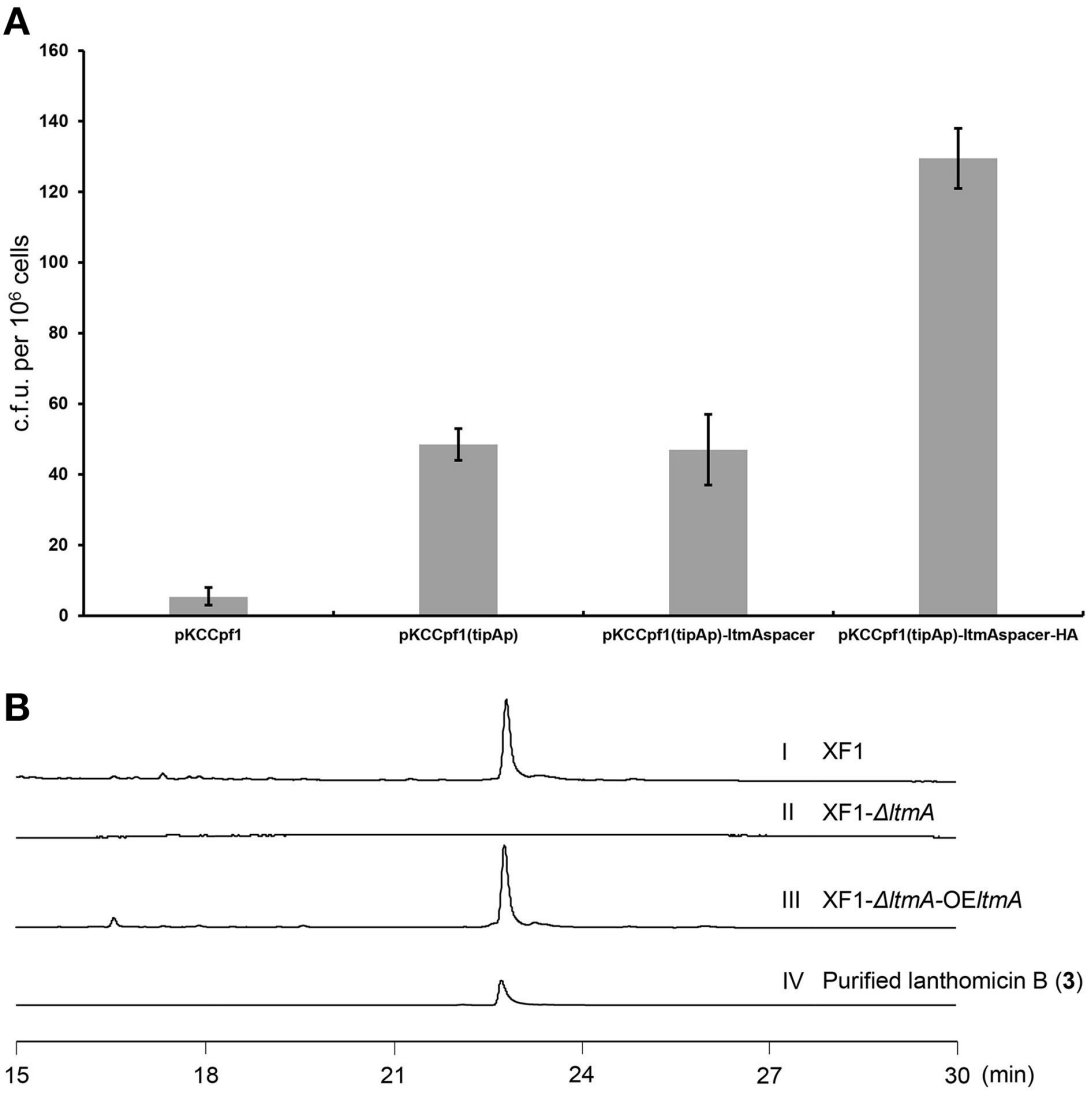
Confirmation of lanthomicins biosynthesis gene cluster *ltm* by the CRISPR-Cpf1-based gene-editing system. **(A)** Transformation efficiency was evaluated by counting the transformant number per 10^6^ viable spores. **(B)** HPLC analysis of lanthomicin B production from *ltmA* deletion and complement strains (UV at 480 nm): (I) *S. chattanoogensis* XF1; (II) *S. chattanoogensis* XF1-ΔA; (III) *S. chattanoogensis* XF1-ΔA-OE-A; and (IV) isolated lanthomicin B.

Traditional genetic manipulation for *S. chattanoogensis* L10 was proved to be intricate and time-consuming (Liu et al., 2015). The CRISPR-Cpf1 system has been widely used in many bacterial species (Meliawati et al., 2021). In the genus *Streptomyces*, this system has been applied successfully to conduct precise genome editing by homology-directed repair (Li et al., 2018). To confirm that the *ltm* gene cluster is responsible for the biosynthesis of the three polyphenol lanthomicins, the functional domain of *ltmA* was used as the target for cleavage. First, the CRISPR-Cpf1 editing plasmid pKCCpf1 was introduced into *S. chattanoogensis* XF1. As Figure 4A shows, the transformation efficiency is very low and we suppose the expression level of Cpf1 is lethal to the host cell. So the inducible promoter *tipAp* was adopted to direct Cpf1 expression. When a gene-specific spacer was introduced into pKCCpf1(*tipAp*) to obtain pKCCpf1(*tipAp*)-ltmAspacer, the target position of *ltmA* in all exconjugants was destroyed, resulting in the abolishment of colored metabolites as compared with the strain XF1 containing pKCCpf1(*tipAp*) (Supplementary Figure 6). The addition of homologous arms (HAs) improved conjugation efficiency significantly and the successfully deleted colonies were found as expected (Figure 4A; Supplementary Figure 7). The metabolic profiles were performed to prove that lanthomicin B was not detected in the resulting strain *S. chattanoogensis* XF1-ΔA by HPLC analysis (Figure 4B II). For the complementation experiment, the *ltmA* gene was randomly inserted into the genome of mutant strain XF1-ΔA affording strain XF1-ΔA-OE-A, which restored the ability to produce lanthomicin B (Figure 4B III). These results suggested that the *ltm* gene cluster was involved in the biosynthesis of lanthomicins.

### Isolation and Characterization of Lanthomicins

**Figure 5:**
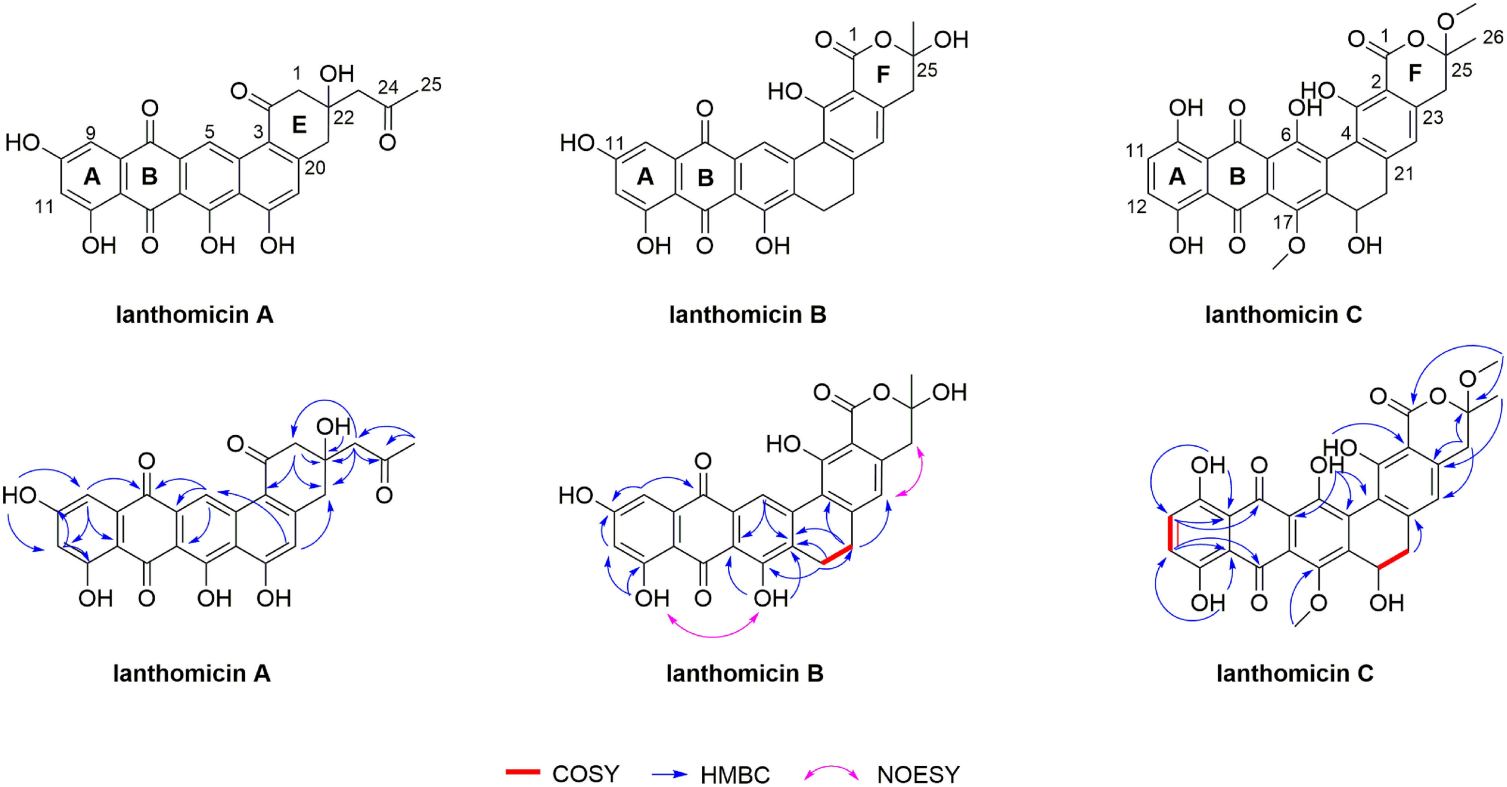
Planar structures of lanthomicin A-C. Key COSY, HMBC, and NOESY correlations of lanthomicin A–C are indicated.

The structure of lanthomicins was elucidated based on NMR data (Supplementary Table 5; Supplementary Figures 13–33). Lanthomicin A (**1**) was obtained as murrey amorphous powder. The molecular formula was determined as C_25_H_18_O_9_ on the basis of the anion peak at *m/z* 461.0879 [M-H]- by high-resolution mass spectrometry (HRMS). Careful comparison of the NMR data of **1** and metabolite 4, which has been activated and isolated from an engineered *Streptomyces viridochromogenes* strain in 2017 (Zhang et al., 2017), revealed that they share a similar dihydrobenzo[α] naphthacenequinone core. The only difference between them was that the methyl group at C-22 in metabolite 4 was replaced by the acetonyl group in **1**. The acetonyl fragment was confirmed by the HMBC correlations from H3-25 (δ 2.17) to C-24 (δ 207.79) and C-23 (δ 53.32). The HMBC correlations from H2-23 (δ 2.68) to C-1 (δ 70.89), C-22 (δ 42.96), and C-2 (δ 52.17) revealed that the acetonyl moiety was attached to C-1. Hence, the structure of **1** was established (Figure 5). HRMS of compound **2** predicted a molecular formula C_26_H_16_O_10_ with exact mass 487.0667 [M-H]-.

Lanthomicin B (**3**) was obtained as a deep orange powder and its molecular formula was C_26_H_18_O_9_ with an anion peak at 473.0876 [M-H]- based on the HRMS data. By literature searching, three main differences existed between **3** and hexaricin F (Gao et al., 2018), the substituents of C-6, C-11, and C-17 were -H, -OH, and -OH in **3** and -OH, -H, and -OCH_3_ in hexaricin F, respectively. The structure was further confirmed by the new peak H-6 (δ 9.02, s) with correlations to C-5 (δ 141.18) and C-7 (δ 130.20), the disappearance of H-11, and the HMBC correlations from OH-17 (δ 12.51) to C-16 (δ 112.88) and C-18 (δ 131.89). Thus, the structure of **3** was established.

Lanthomicin C (4) was obtained as scarlet amorphous powder with molecular formula C_28_H_22_O_11_ according to the anion peak at *m/z* 533.1087 [M-H]-. The COSY correlations of H-11 (7.30, d)/H-12 (7.34, d) indicated the difference in ring A between **3** and **4**, and this was further confirmed by the HMBC correlations from HO-10 (δ 12.14) to C-9 (δ 113.42) and C-11 (δ 129.29). Another main COSY difference occurred in H-19 (δ 4.68)/H-20 (δ 3.51, 2.53), the lack of one hydrogen confirmed the oxhydryl introduced at C-19. And for **4**, two extra molecules of methyl were linked by HMBC correlations from δ 3.41 to C-1 (δ 170.18) and C-25 (δ 106.72), from δ 3.89 to C-17 (δ 152.57). Henceforth, the structure of **4** was finished.

### Antitumor Bioactivity of Lanthomicins

It has been reported that compounds synthesized by type II PKS often showed better inhibitory activity against tumor cell lines (Gan et al., 2015; Woo et al., 2016; Jiang et al., 2018). Therefore, we performed *in vitro* cytotoxicity assays for lanthomicins toward cancer cell lines using doxorubicin as a positive control. The results indicated that lanthomicin A showed antiproliferative activity toward lung cancer cell line A-549 with IC_50_ 0.17 μM, while lanthomicin B and C demonstrated nonsignificant antiproliferative activity against all the cell lines (Table 2; Supplementary Figure 8).

**Table 2:** Antitumor activity of lanthomicins.

**Tumor cell lines**	**IC**_**50**_ **(μM/L)**			
	**Doxorubicin**	**Lanthomicin A**	**Lanthomicin B**	**Lanthomicin C**
A-549 (lung)	0.02	0.17	>100	>100
MCF-7 (breast)	0.62	5.98	>100	>100
HepG2 (liver)	4.38	34.34	>100	>100
HCT-116 (colon)	10.58	82.27	>100	>100

### Proposal of a Biosynthetic Pathway for Lanthomicins

**Figure 6:**
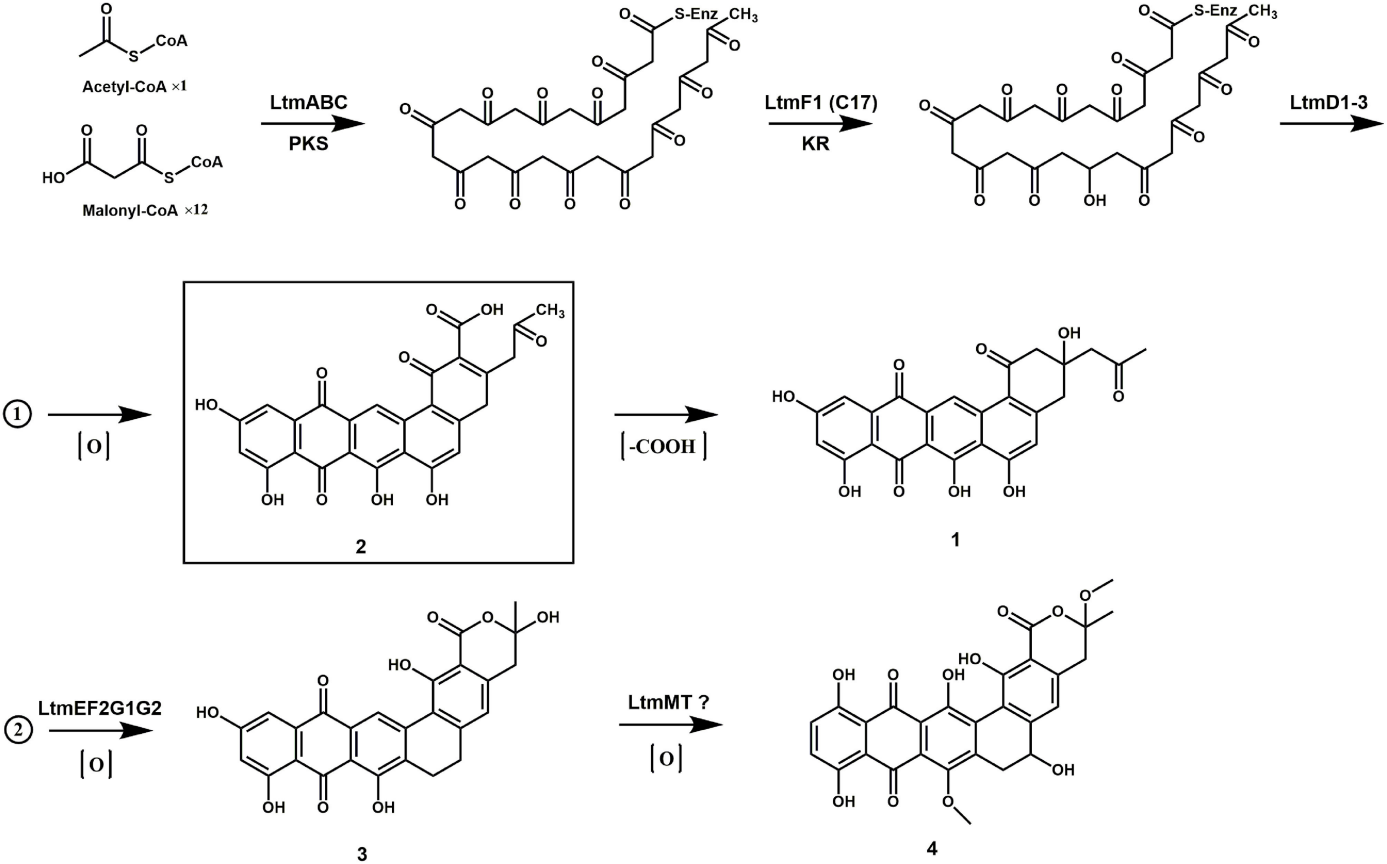
The proposed biosynthetic pathway of lanthomicins.

The biosynthesis of the lanthomicins can be rationalized on the basis of successive activation of silent genes and functional analysis by homologous comparison. The key enzymes predicted to be involved in this process are shown in Figure 1C. The biosynthesis probably begins with condensing malonate units by the minimal type II PKSs LtmABC (Ketosynthase, KS_α_; chain length factor or KS_β_; ACP). Three enzymes LtmH1-H3 seem to be responsible for the biosynthesis of malonyl-CoA from the carboxylation of acetyl-CoA. Three KRs were found in *ltm* cluster and a phylogenetic analysis implied that LtmF1 might belong to C17 KRs, while LtmF2 was thus the likely C19 KR (Supplementary Figure 9). Thus LtmF1 was probably involved in the reduction of the keto group at C17 of the nascent polyketide chain to a hydroxyl group. The cyclases LtmD1-D3 were supposed to further modify the reduced skeleton directing the cyclization through intramolecular aldol condensations to form the angular 5-ring aromatic structure. Activation of the multicistronic cassette *ltmF1D1D2ABCD3* could produce lanthomicin A and compound **2** (Figure 6 route 1). Three monooxygenases LtmE, LtmG1-G2, and a predicted C19 KR LtmF2 were also activated by promoter substitution and the fermentation profile changed leading to the discovery of lanthomicin B. LtmF2 was thus likely to catalyze the reduction of the 19-ketone to a hydroxyl group. We proposed that three monooxygenases might take responsibility for the closure of ring F in lanthomicin B. Lanthomicin C showed more methylation and hydroxylation than lanthomicin B. LtmF3, which was predicted as a C11 KR, thus might catalyze reduction in C11 carbonyl. LtmJ showed 55% identity to FdmM, which hydroxylated the C6 site during the biosynthesis of fredericamycin A (Chen et al., 2009), might also play a similar role during the biosynthesis (Figure 6 route 2).

## Discussion

Secondary metabolites embedded in cryptic biosynthetic gene clusters (BGCs) with potential clinical value are well belongings of filamentous microorganisms, especially the genus *Streptomyces*. To activate cryptic clusters in *S. chattanoogensis* L10, we consider that natamycin is the main product of this strain and that blocking the main products in *Streptomyces* accelerates new compounds discovery (Culp et al., 2019). So we knocked out the first PKS gene in natamycin BGC to block its production, and we found that no new secondary metabolites were produced. We then used the natamycin-inactivated strain for activation. As mentioned above, 11 putative BGCs containing PKSs or NRPSs were found in the genome of *S. chattanoogensis* L10. Except for natamycin, chattamycin, and lanthomicin gene clusters, we also activated cluster 20 by a regulatory means. Recently, azoxymycins were found to be synthesized by linking two precursors with an azoxy bond (Guo et al., 2015) and how this unusual chemical connection could be formed was deeply interpreted (Guo et al., 2019). Several terpene and polypeptide BGCs were also found and bioactive polypeptide antibiotics could be identified by uncovering these cryptic pathways (Daniel-Ivad et al., 2017).

In this work, we found the *ltm* gene cluster with homology to *xan* and *arx* gene clusters, the corresponding natural products belong to the pentangular polyphenols subclade of aromatic PKS. The chemical diversity of this subclade is dependent on the postsynthetic modifications by some enzymes like methyltransferase and glycosyltransferase, especially some unknown oxygenases. It is worth mentioning that the pentangular polyphenols often display an inhibitory effect on tumor cells (Winter et al., 2013). To activate the cryptic *ltm* gene cluster, we adopt a combined strategy. We first overexpressed the putative positive regulator, disrupted the repressor gene, and combined the both. However, all these attempts failed to activate the gene cluster. Then we constitutively expressed the core gene cassette, resulting in the production of lanthomicin A and compound **2**. The further knock-in of strong promoter kasO^*^p in the upstream of *ltmF1* along with changing fermentation environment ultimately led to the discovery of lanthomicin B and lanthomicin C. QRT-PCR showed that the transcriptional levels from *ltmF1* to *ltmH3* were strikingly improved and these genes seemed to be cotranscribed. Then, we successfully applied the CRISPR-Cpf1-based gene-editing tool to knock out the core skeleton gene *ltmA* in *ltm* gene cluster. The *ltmA* gene knockout and complement experiments further confirmed that the *ltm* gene cluster is responsible for the biosynthesis of the three polyphenol lanthomicins.

In this study, three novel pentangular polyphenols lanthomicin A–C were characterized by structure elucidation and antiproliferative activity evaluation. Lanthomicin A showed a characteristic in the E ring that the carboxyl group seemed to be lost and this situation was also found in the biosynthesis of pradimicin A (Zhan et al., 2008). Lanthomicin A also showed antiproliferative activity toward all of the tested cancer cell lines, with IC_50_ values of 0.17 and 5.98 μM in A-549 and MCF-7 cells, respectively. Lanthomicin C showed more methylation and hydroxylation than others and reduction in C11 carbonyl was assumed to be catalyzed by the predicted C11 KR LtmF3 under a changed fermentation environment. There are no clues about which enzymes may be responsible for the formation of bonds at C5-C18 and C2-C23 (C1-C2 for lanthomicin A). Zhan and coworkers showed a monooxygenase PdmH worked collaboratively with two cyclases to form the 5-ring structure (Zhan et al., 2008). Hence, further studies were needed to uncover the cyclization in lanthomicin biosynthesis.

## Data Availability Statement

The datasets presented in this study can be found in online repositories. The names of the repository/repositories and accession number(s) can be found in the article/Supplementary Material.

## Author Contributions

X-FL: conceptualization, methodology, validation, investigation, data curation, formal analysis, visualization, writing—original draft, and writing—review and editing. J-XW: methodology, validation, and formal analysis. X-AC: formal analysis and writing—review and editing. YL: data curation and formal analysis. Y-QL: conceptualization, project administration, funding acquisition, supervision, and writing—review and editing. All authors read and approved the final manuscript.

## Funding

This work was supported by the National Key Research and Development Program (Nos. 2018YFA0903200 and 2019YFA09005400) and the National Natural Science Foundation of China (Nos. 31730002 and 31520103901).

## Conflict of Interest

The authors declare that the research was conducted in the absence of any commercial or financial relationships that could be construed as a potential conflict of interest.

## Publisher's Note

All claims expressed in this article are solely those of the authors and do not necessarily represent those of their affiliated organizations, or those of the publisher, the editors and the reviewers. Any product that may be evaluated in this article, or claim that may be made by its manufacturer, is not guaranteed or endorsed by the publisher.
