# Supplementary material for: Activation and Characterization of Lanthomicins A–C by Promoter Engineering in Streptomyces chattanoogensis L10
Source: Front Microbiol. 2022 May 10;13:902990. doi: 10.3389/fmicb.2022.902990 (PMC9127795; doi:10.3389/fmicb.2022.902990)
Supplement: Supplementary file 1 [file Table_1.DOCX]

Supplementary Table S1. Strains used in this work.

| Strains | Description | | Sourse |
| --- | --- | --- | --- |
| ***Streptomyces*** | |  |  |
| *S. chattanoogensis* L10 | | Three BGCs were disrupted in *S. chattanoogensis* L10 |  |
| *S. chattanoogensis* L10-OE-R1 | | *ltmR1* gene overexpression strain | This study |
| *S. chattanoogensis* L10-ΔR2 | | *ltmR2* gene deletion strain | This study |
| *S. chattanoogensis* L10-OE-F1D3 | | *ltmF1-D3* gene overexpression strain | This study |
| *S. chattanoogensis* XF1 | | Native promoter of *ltmF1* was replaced by *kasO** promoter in *S. chattanoogensis* L10 | This study |
| *S. chattanoogensis* XF1-ΔA | | Functional domain of *ltmA* was removed by in-frame deletion in *S. chattanoogensis* XF1 | This study |
| *S. chattanoogensis* XF1-ΔA-OE-A | | Compensatory strain by introducing an extra copy of *ltmA* gene in *S. chattanoogensis* XF1-ΔA | This study |
| ***E. coli*** | |  |  |
| *E. coli* DH5α | | For gene cloning |  |
| *E. coli* ET12567/pUZ8002 | | For inter-species plasmid transfering |  |
|  | |  |  |
