# Supplementary material for: Activation and Characterization of Lanthomicins A–C by Promoter Engineering in Streptomyces chattanoogensis L10
Source: Front Microbiol. 2022 May 10;13:902990. doi: 10.3389/fmicb.2022.902990 (PMC9127795; doi:10.3389/fmicb.2022.902990)
Supplement: Supplementary file 2 [file Table_2.DOCX]

Supplementary Table S2. Plasmids used in this work.

| Plasmids | Description | Source |
| --- | --- | --- |
| pSET152-spec | *aac*(3)IV was replaced by *aadA* in pSET152 | This study |
| pKC1139-spec | *aac*(3)IV was replaced by *aadA* in pKC1139 | This study |
| pSET152-spec-*kasO** | General vector for gene overexpression | This study |
| pSET152-spec-*kasO**-ltmR1 (pXF01) | For *ltmR1* gene overexpression | This study |
| pKC1139-spec-UHA-ltmR2-DHA (pXF02) | For *ltmR2* gene deletion | This study |
| pSET152-spec-*kasO**-ltmF1D1D2ABCD3 (pXF03) | For multicistronic cassette overexpression | This study |
| pKC1139-spec-UHA-Km*kasO**p-DHA (pXF04) | For promoter substitution | This study |
| pKCCpf1(*tipA*p) |  | (Li et al. 2018) |
| pKCCpf1(*tipA*p)-ltmAspacer (pXF05) | For *ltmA* gene disruption | This study |
| pKCCpf1(*tipA*p)-ltmAspacer-HA (pXF06) | For precise *ltmA* gene deletion by homologous recombination | This study |
| pSET152-spec-*kasO**-ltmA (pXF07) | For compensatory function of *ltmA* | This study |

**References**

Li, L., Wei, K. K., Zheng, G. S., Liu, X. C., Chen, S. X., Jiang, W. H., *et al*. (2018). CRISPR-Cpf1-assisted multiplex genome editing and transcriptional repression in *Streptomyces*. *Appl. Environ. Microbiol*. 84, e00827-18. doi: 10.1128/AEM.00827-18.
