# Supplementary material for: Activation and Characterization of Lanthomicins A–C by Promoter Engineering in Streptomyces chattanoogensis L10
Source: Front Microbiol. 2022 May 10;13:902990. doi: 10.3389/fmicb.2022.902990 (PMC9127795; doi:10.3389/fmicb.2022.902990)
Supplement: Supplementary file 3 [file Table_3.DOCX]

Supplementary Table S3. Primers used in this work.

| Primers | Sequences (5’-3’)^a^ |
| --- | --- |
| spec-F | aatacgaatggcgaaaagccGTTCATGTGCAGCTCCATC |
| spec-R | tcatctcgttctccgctcatTTATTTGCCGACTACCTTG |
| *kasO**p-F | gggctgcaggtcgactctagaTGTTCACATTCGAACCGTCTCT |
| *kasO**p-R | ctatgacatgattacgaattcagatctcatatgAACTCCCCCAGTCCTGCAC |
| ltmR1-F | cgtgcaggactgggggagttGTGCAAACCTCGGAACTCAT |
| ltmR1-R | catgattacgaattcagatctTCAGGTGAGGTTCTTCACCC |
| delltmR2-SF | acgacggccagtgccaagcttCGGTCATTGCGTAGACACTC |
| delltmR2-SR | cggtcgttgccgcctgacatCGAAGACACAAGACGACTATACAC |
| delltmR2-XF | ATGTCAGGCGGCAACGAC |
| delltmR2-XR | catgattacgaattcgatatcTGCGGATCATCCACTACGAG |
| cassatte-F1 | cgtgcaggactgggggagttGTGGCCTGCGAACTCGC |
| cassatte-R1 | CTGCTTGGTACCGGAGCC |
| cassatte-F2 | CGACGTGGATTACATCAATGC |
| cassatte-R2 | tatgacatgattacgaattcGCTCCATCCCTCAAGCCTC |
| ltmF1-SF | cgggcgcaagccgccaagcttGCGGGCGATCTGGTCTTG |
| ltmF1-SR | tttcgtcaaaaacctggCCCCGGTGATCAGTGCAGT |
| Km*kasO**p-F | CCAGGTTTTTGACGAAATGGATC |
| *kasO**p-R2 | AACTCCCCCAGTCCTGCAC |
| ltmF1-XF | cgtgcaggactgggggagttGTGGCCTGCGAACTCGC |
| ltmF1-XR | catgattacgaattcgatatcTCCTCGGAGTACGGGTGGTAG |
| ltmAspacer-F | cgagatatcgacgc*gccacggtgtcacgctgatcgag*ATCTACAACAGTAGAAATTTGG |
| ltmAspacer-R | ctcctggtagatggacatatgTCCGCTCCCTTCTCTGACG |
| delltmA-SF | atcaagggtacctacgagatGTGGCCTGCGAACTCGC |
| delltmA-SR | cgcgcggcctcggcctcttcGGCTGTCCTGCTCATCGAG |
| delltmA-XF | GAAGAGGCCGAGGCCGC |
| delltmA-XR | tgacaccgtggcgcgtcgatGCCTCGGTCAGATCGTCCTC |
| ltmA-F | cgtgcaggactgggggagttATGAGCAGGACAGCCGCT |
| ltmA-R | tacgaattcagatctcatatgTCAGCTGCACGCCCTTTC |

^a^Primer sequences are indicated with capital letter; Homologous arm sequences are indicated with lowercase; The restriction sites are underlined; 23nt spacer sequence is colored and in italics.
