# Supplementary material for: Activation and Characterization of Lanthomicins A–C by Promoter Engineering in Streptomyces chattanoogensis L10
Source: Front Microbiol. 2022 May 10;13:902990. doi: 10.3389/fmicb.2022.902990 (PMC9127795; doi:10.3389/fmicb.2022.902990)
Supplement: Supplementary file 4 [file Table_4.DOCX]

Supplementary Table S4. AntiSMASH-predicted BGCs for *Streptomyces chattanoogensis* L10.

| BGC | Position | | Product type | Compound | Study |
| --- | --- | --- | --- | --- | --- |
|  | From | To |  |  |  |
| Cluster 1 | 789 | 55581 | T2 PKS | Lanthomicins | This study |
| Cluster 2 | 63109 | 82363 | Terpene |  |  |
| Cluster 3 | 118159 | 128629 | Melanin |  |  |
| Cluster 4 | 161543 | 183742 | Unknown |  |  |
| Cluster 5 | 226143 | 246544 | Terpene |  |  |
| Cluster 6 | 595831 | 618578 | Lanthipeptide |  |  |
| Cluster 7 | 713451 | 758838 | T1 PKS |  |  |
| Cluster 8 | 779128 | 826411 | Terpene |  |  |
| Cluster 9 | 915447 | 936694 | Unknown |  |  |
| Cluster 10 | 1016277 | 1087545 | T2 PKS |  |  |
| Cluster 11 | 1264783 | 1284300 | Terpene |  |  |
| Cluster 12 | 1313388 | 1333013 | CDPS |  |  |
| Cluster 13 | 1337432 | 1378583 | Unknown |  |  |
| Cluster 14 | 1575079 | 1585213 | Butyrolactone |  |  |
| Cluster 15 | 1713129 | 1732772 | Terpene |  |  |
| Cluster 16 | 1804527 | 1890375 | TransAT-PKS |  |  |
| Cluster 17 | 1920301 | 1978484 | Lassopeptide |  |  |
| Cluster 18 | 2247879 | 2261794 | Siderophore |  |  |
| Cluster 19 | 2407720 | 2487623 | T2 PKS | Chattamicins | Zhou et al., 2015 |
| Cluster 20 | 2825953 | 2904962 | T1 PKS | Sansimycin | Unreported |
| Cluster 21 | 2983660 | 2992398 | Siderophore |  |  |
| Cluster 22 | 3073690 | 3084106 | Ectoine |  |  |
| Cluster 23 | 3611027 | 3631866 | Lassopeptide |  |  |
| Cluster 24 | 3949807 | 3969125 | Terpene |  |  |
| Cluster 25 | 5533913 | 5556150 | Terpene |  |  |
| Cluster 26 | 7284332 | 7299020 | Siderophore |  |  |
| Cluster 27 | 7414177 | 7425124 | Butyrolactone |  |  |
| Cluster 28 | 7557609 | 7608322 | NRPS |  |  |
| Cluster 29 | 7923360 | 7947921 | Lanthipeptide |  |  |
| Cluster 30 | 7965501 | 8006565 | T3 PKS |  |  |
| Cluster 31 | 8155284 | 8176399 | Cyanobactin |  |  |
| Cluster 32 | 8177764 | 8231876 | T1 PKS-NRPS |  |  |
| Cluster 33 | 8311787 | 8430478 | T1 PKS | Natamycin | Du et al. 2009 |
| Cluster 34 | 8448974 | 8469687 | CDPS |  |  |
| Cluster 35 | 8638838 | 8696434 | NRPS |  |  |
| Cluster 36 | 8708354 | 8754118 | Lassopeptide |  |  |
| Cluster 37 | 9010346 | 9034129 | Lanthipeptide |  |  |

**References**

Du, Y. L., Chen, S. F., Cheng, L. Y., Shen, X. L., Tian, Y., and Li, Y. Q. (2009). Identification of a novel *Streptomyces chattanoogensis* L10 and enhancing its natamycin production by overexpressing positive regulator ScnRII. *J. Microbiol*. 47, 506-13. doi: 10.1007/s12275-009-0014-0.

Zhou, Z. X., Xu, Q. Q., Bu, Q. T., Guo, Y. Y., Liu, S. P., Liu, Y., *et al*. (2015). Genome mining-directed activation of a silent angucycline biosynthetic gene cluster in *Streptomyces chattanoogensis*. *ChemBioChem*. 16, 496-502. doi: 10.1002/cbic.201402577.
