# Supplementary material for: Activation and Characterization of Lanthomicins A–C by Promoter Engineering in Streptomyces chattanoogensis L10
Source: Front Microbiol. 2022 May 10;13:902990. doi: 10.3389/fmicb.2022.902990 (PMC9127795; doi:10.3389/fmicb.2022.902990)
Supplement: Supplementary file 5 [file Table_5.DOCX]

Supplementary Table S5. ^1^H (600 MHz) and ^13^C (150 MHz) NMR data of lanthomicin A(**1**), B(**3**) and C(**4**)

| **position** |  | **1** (DMSO-*d*_6_) | |  | **3** (DMSO-*d*_6_) | | |  | **4** (acetone-*d*_6_) | |
| --- | --- | --- | --- | --- | --- | --- | --- | --- | --- | --- |
|  |  | *δ*_H_ | *δ*_C_ |  | *δ*_H_ | *δ*_C_ | |  | *δ*_H_ | *δ*_C_ |
| 1 |  |  | 70.8 |  |  | | 171.7 |  |  | 170.1 |
| 1-OH |  | 5.05(1H, brs) |  |  |  | |  |  |  |  |
| 2 |  | 2.62(1H, s) | 52.1 |  |  | | 108.8 |  |  | 107.8 |
|  |  | 2.85(1H, s) |  |  |  | |  |  |  |  |
| 3 |  |  | 195.8 |  |  | | 163.1 |  |  | 160.0 |
| 3-OH |  |  |  |  |  | |  |  | 12.01(1H, brs) |  |
| 4 |  |  | 150.1 |  |  | | 118.5 |  |  | 115.0 |
| 5 |  |  | 137.0 |  |  | | 141.1 |  |  | 130.6 |
| 6 |  | 9.34(1H, s) | 115.4 |  | 9.02(1H, s) | | 119.8 |  |  | 158.4 |
| 6-OH |  |  |  |  |  | |  |  | 13.42(1H, brs) |  |
| 7 |  |  | 135.4 |  |  | | 130.2 |  |  | 114.5 |
| 8 |  |  | 183.3 |  |  | | 181.5 |  |  | 191.8 |
| 9 |  |  | 132.8 |  |  | | 135.4 |  |  | 113.4 |
| 10 |  | 7.06(1H, s) | 105.8 |  | 7.16(1H, d, 2.3) | | 108.8 |  |  | 158.6 |
| 10-OH |  |  |  |  |  | |  |  | 12.14(1H, brs) |  |
| 11 |  |  | 162.9 |  |  | | 165.6 |  | 7.30(1H, d, 9.3) | 129.2 |
| 11-OH |  |  |  |  | 11.44(1H, brs) | |  |  |  |  |
| 12 |  | 6.51(1H, s) | 108.0 |  | 6.60(1H, d, 2.2) | | 107.8 |  | 7.34(1H, d, 9.3) | 130.5 |
| 13 |  |  | 164.4 |  |  | | 164.5 |  |  | 157.8 |
| 13-OH |  |  |  |  | 12.15(1H, s) | |  |  | 13.38(1H, brs) |  |
| 14 |  |  | 111.2 |  |  | | 109.2 |  |  | 114.4 |
| 15 |  |  | 184.4 |  |  | | 189.5 |  |  | 186.8 |
| 16 |  |  | 108.5 |  |  | | 112.8 |  |  | 123.6 |
| 17 |  |  | 169.9 |  |  | | 157.9 |  |  | 152.5 |
| 17-OH |  |  |  |  | 12.51(1H, s) | |  |  |  |  |
| 17-OCH_3_ |  |  |  |  |  | |  |  | 3.89(3H, d, 3.8) |  |
| 18 |  |  | 118.7 |  |  | | 131.8 |  |  | 143.9 |
| 19 |  |  | 173.9 |  | 2.83(overlap) | | 20.1 |  | 4.68(1H, s) | 68.6 |
| 19-OH |  |  |  |  |  | |  |  | 5.07(1H, s) |  |
| 20 |  | 6.42(1H, s) | 113.4 |  | 2.72(overlap) | | 28.3 |  | 3.51(1H, s) | 41.3 |
|  |  |  |  |  |  | |  |  | 2.53(1H, s) |  |
| 21 |  |  | 116.7 |  |  | | 143.5 |  |  | 143.9 |
| 22 |  | 2.98(1H,s)3.23(1H, s) | 42.9 |  | 6.45(1H, s) | | 120.2 |  | 7.20(1H, s) | 114.4 |
| 23 |  | 2.68(1H, s) | 53.3 |  |  | | 140.5 |  |  | 141.1 |
| 24 |  |  | 207.7 |  | 3.17(1H, s) | | 48.6 |  | 3.31(1H, s) | 39.2 |
|  |  |  |  |  | 3.17(1H, s) | |  |  | 3.46(1H, s) |  |
| 25 |  | 2.17(1H, s) | 32.4 |  |  | | 107.8 |  |  | 106.7 |
| 25-OH |  |  |  |  | 4.16(1H, brs) | |  |  |  | 50.5 |
| 25-OCH_3_ |  |  |  |  |  | | 29.1 |  | 3.41(3H, s) | 22.8 |
| 26 |  |  |  |  | 2.07(1H, s) | |  |  | 1.74(1H, s) |  |
