# Supplementary material for: Activation and Characterization of Lanthomicins A–C by Promoter Engineering in Streptomyces chattanoogensis L10
Source: Front Microbiol. 2022 May 10;13:902990. doi: 10.3389/fmicb.2022.902990 (PMC9127795; doi:10.3389/fmicb.2022.902990)
Supplement: Supplementary file 6 [file Data_Sheet_1.docx]

**Supplementary Figures S1-S33**


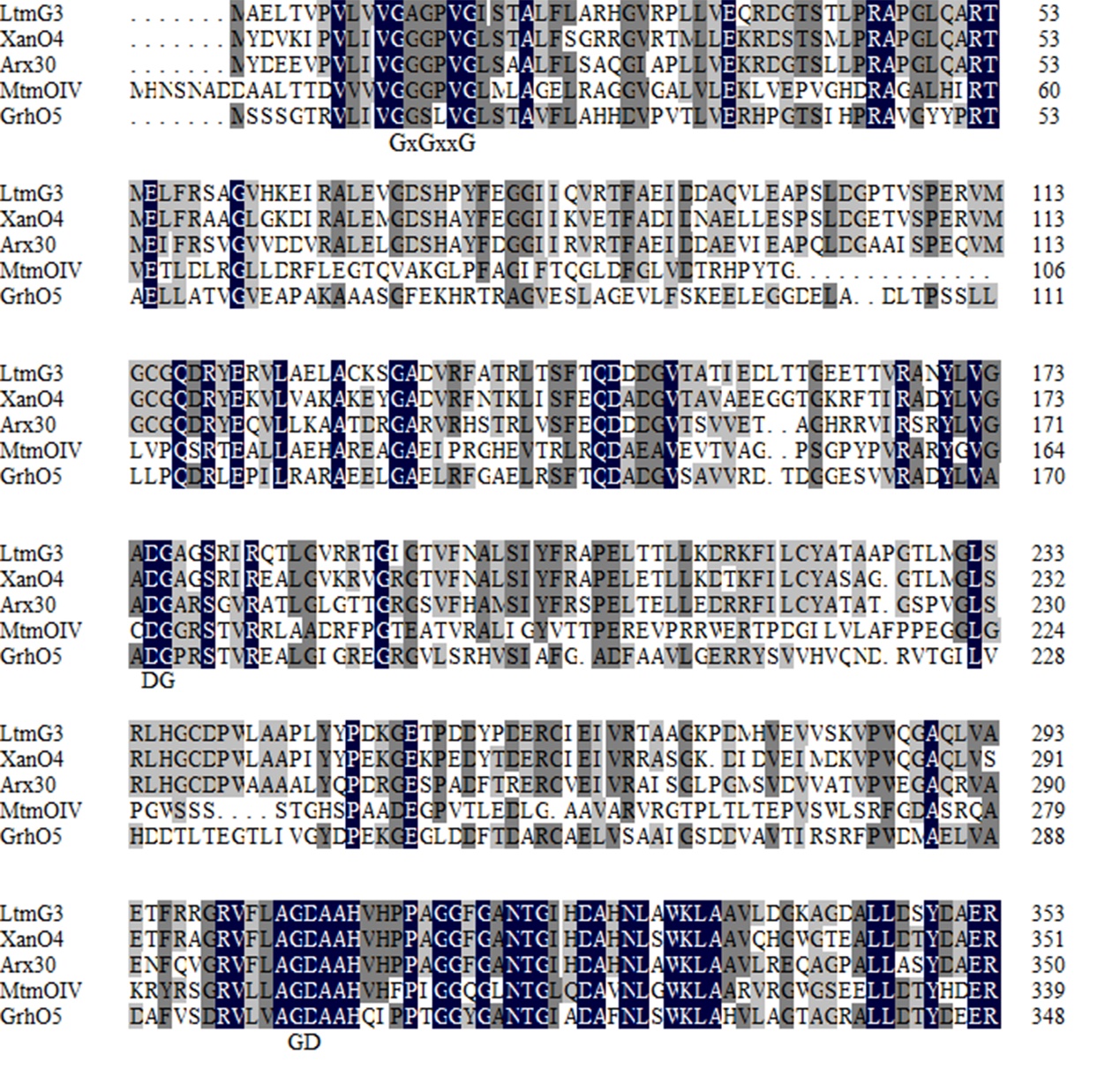


Supplementary Figure S1. Multiple sequence alignment of LtmG3 with other BVMO homologous proteins. The residues conserved in active sites are marked. GeneBank accession numbers for each of the enzymes are XanO4, ADE22300; Arx30, AHX24751; MtmOIV, CAK50794; GrhO5, AAM33672.


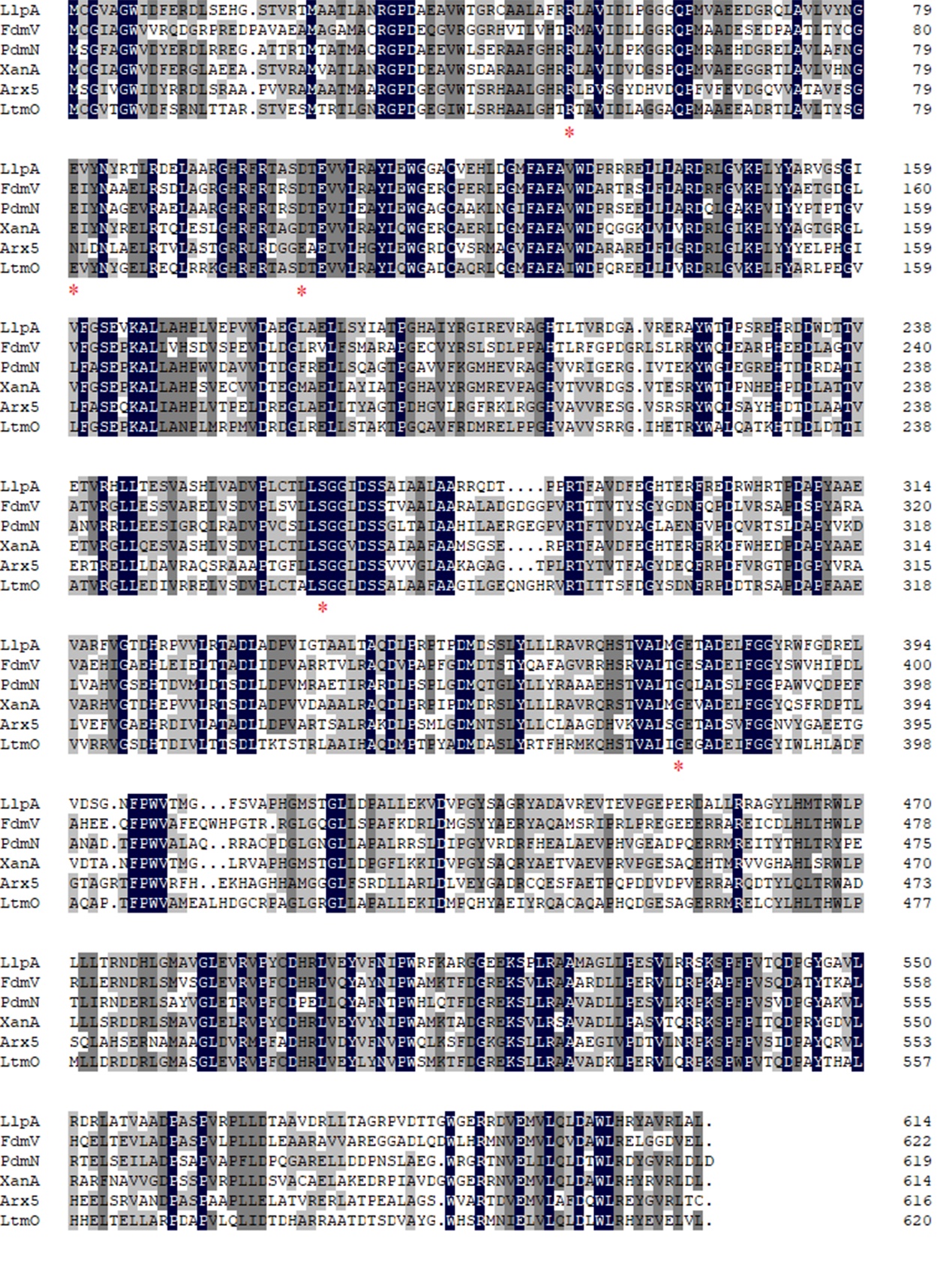


Supplementary Figure S2. Multiple sequence alignment of LtmO with its homologs. The conserved amino acid sites for ligand binding and catalyzing are marked with red asterisks.


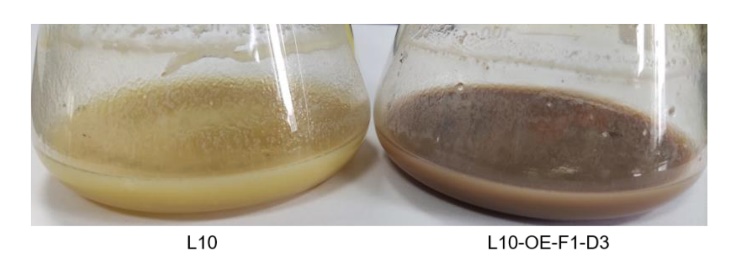


Supplementary Figure S3. Lanthomicins were produced in liquid YEME flask by overexpression of *ltmF1-D3* cassette.


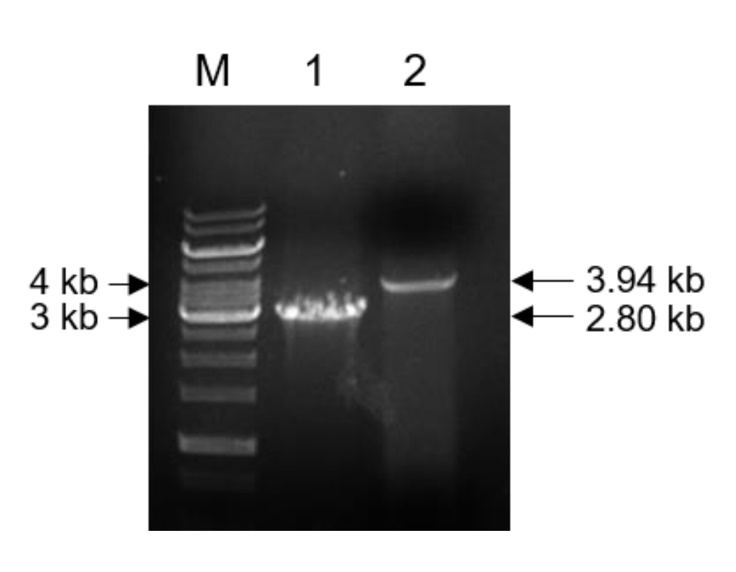


Supplementary Figure S4. PCR verification of replaced mutant. M: Marker; 1: *S. chattanoogensis* L10; 2: *S. chattanoogensis* XF1.


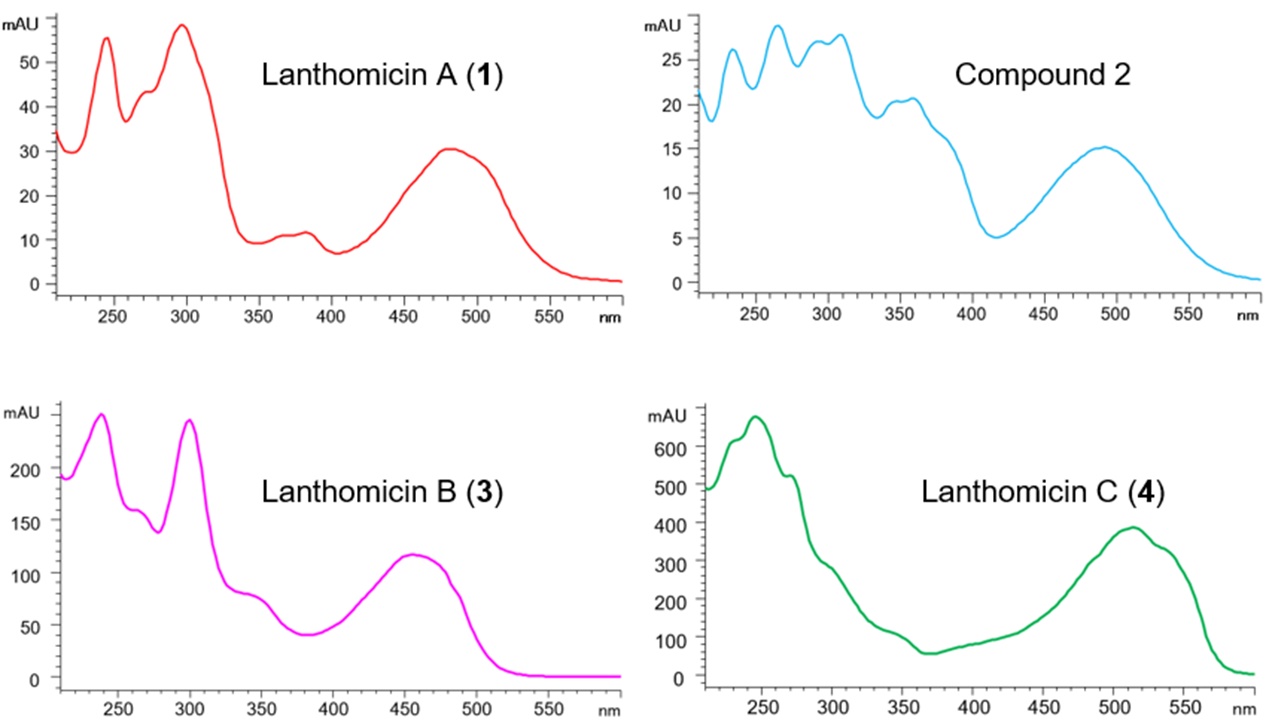


Supplementary Figure S5. UV spectra of Lanthomicins.


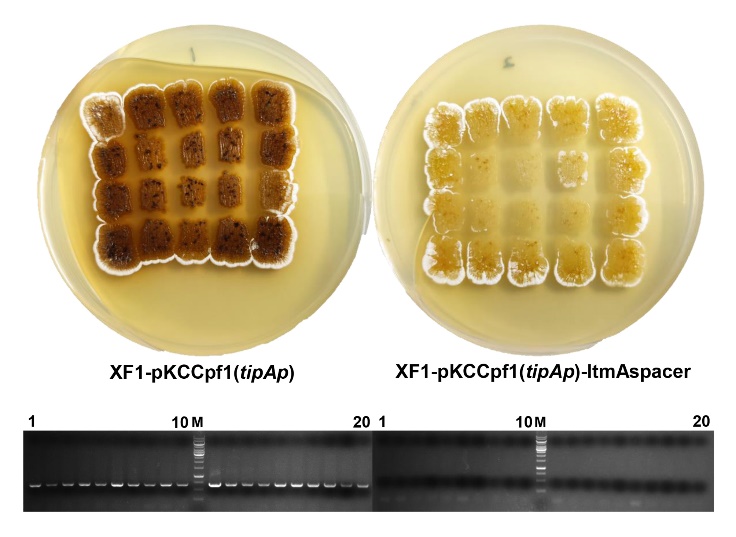


Supplementary Figure S6. Phenotypic and genotypic verification of random selected exconjugants containing plasmid pKCCpf1(*tipAp*) or pKCCpf1(*tipAp*)-ltmAspacer.


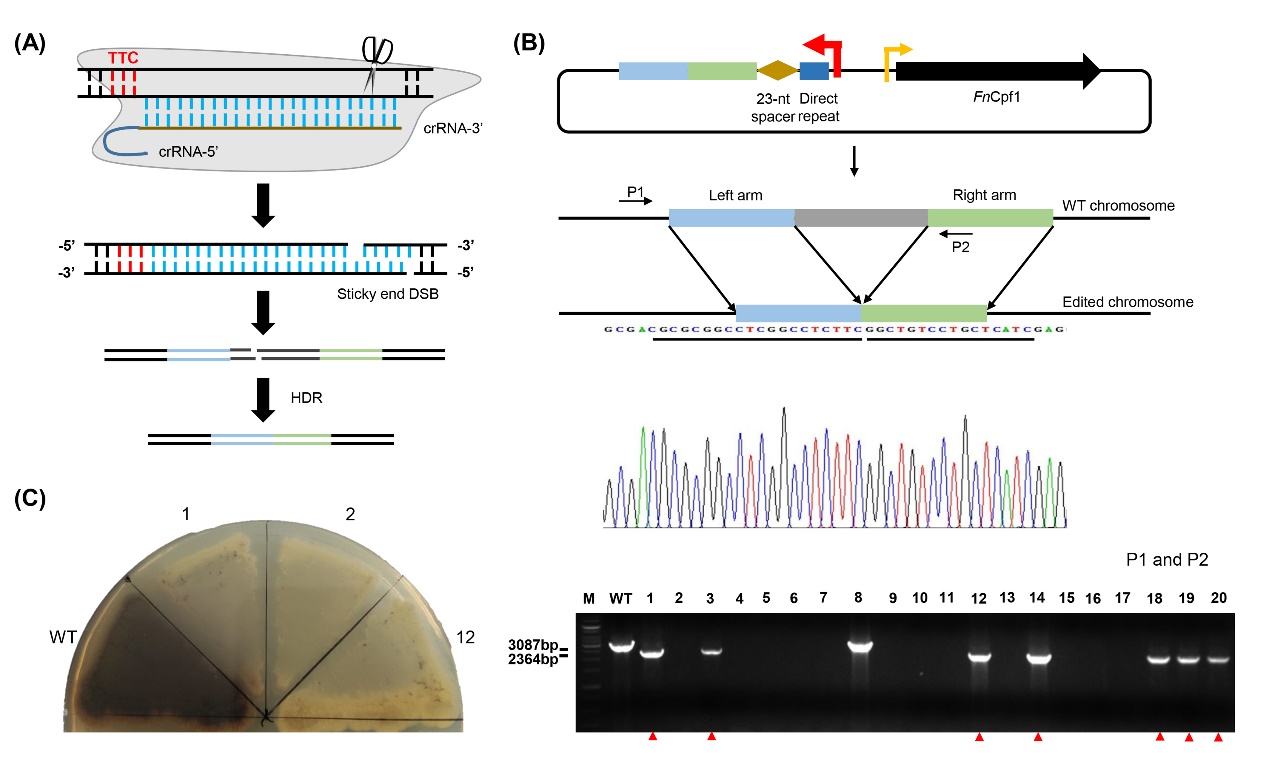


Supplementary Figure S7. CRISPR-Cpf1 assisted gene editing. **(A)** Overview of the Cpf1 system in gene in-frame deletion. **(B)** Schematic of *ltm* structural gene editing using Cpf1 with a single spacer cassette and an editing template. **(C)** Phenotypic evaluation and PCR evaluation of the starting strain *S. chattanoogensis* XF1 and 20 randomly selected exconjugants.


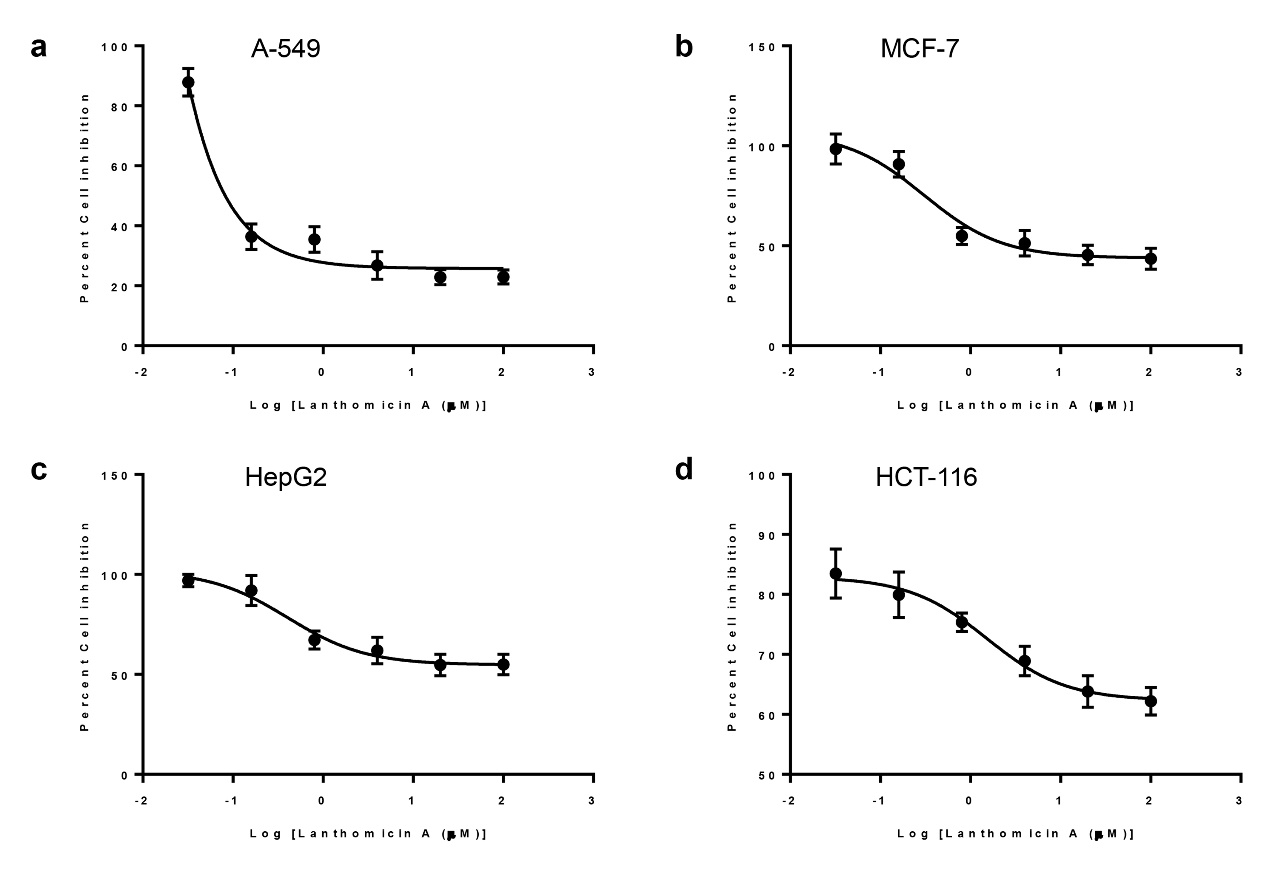


Supplementary Figure S8. Anti-proliferative activity of lanthomicin A.





Supplementary Figure S9. Phylogenetic analysis of LtmF1, LtmF2, LtmF3 and their homologs.


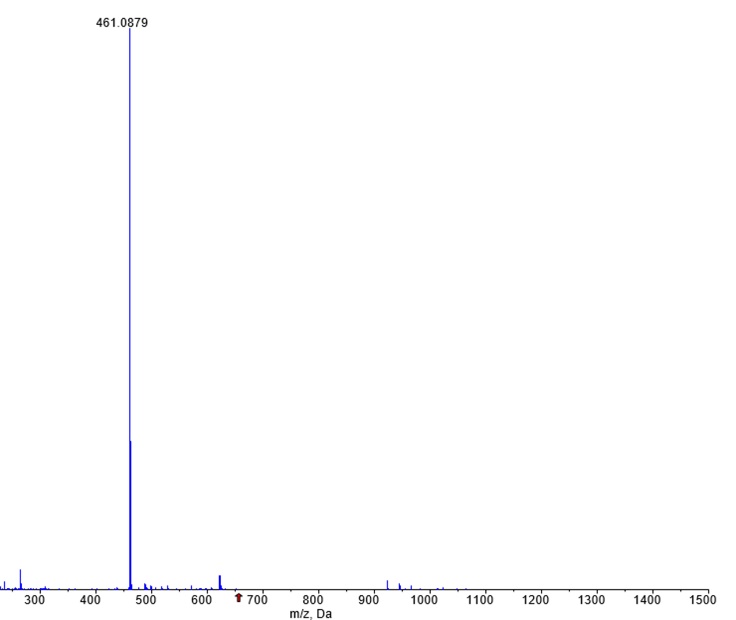


Supplementary Figure S10. HR-ESI-MS spectra of Lanthomicin A (compound **1**).

**
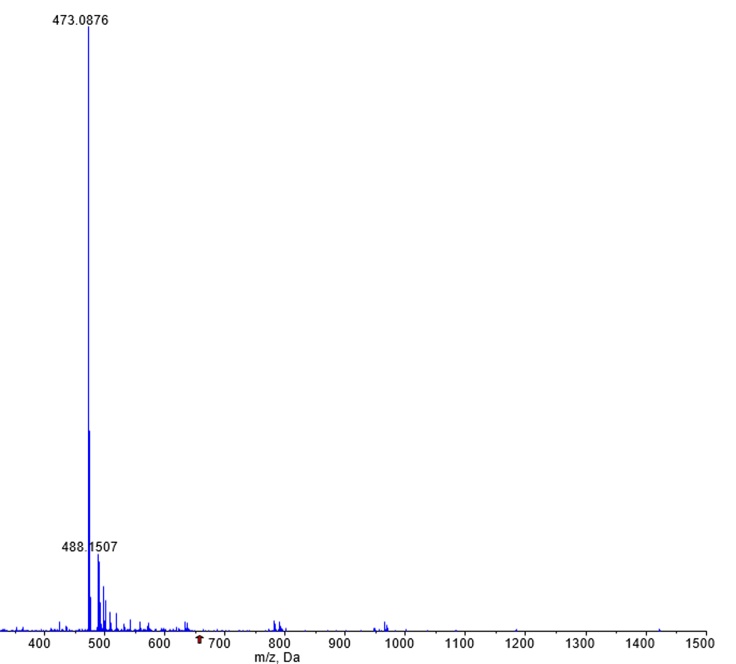
**

Supplementary Figure S11. HR-ESI-MS spectra of Lanthomicin B (compound **3**).


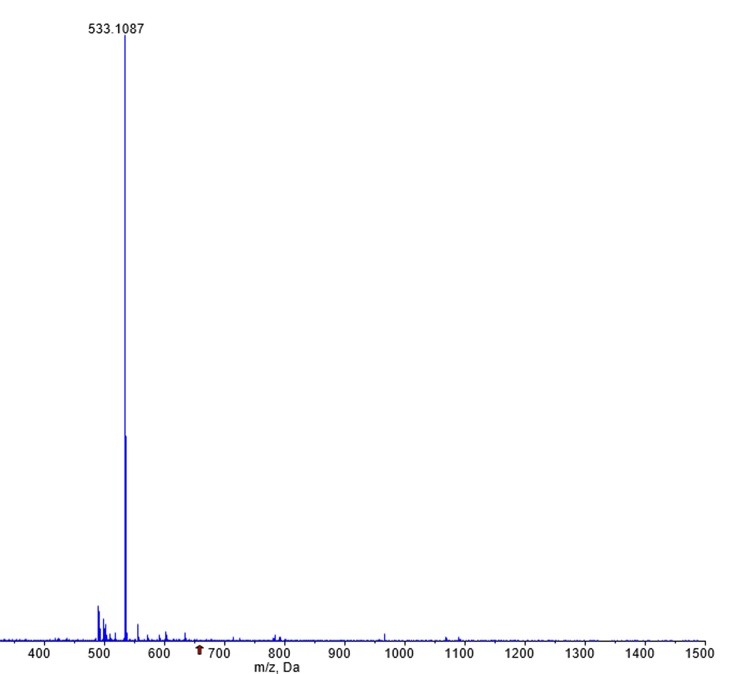


Supplementary Figure S12. HR-ESI-MS spectra of Lanthomicin C (compound **4**).


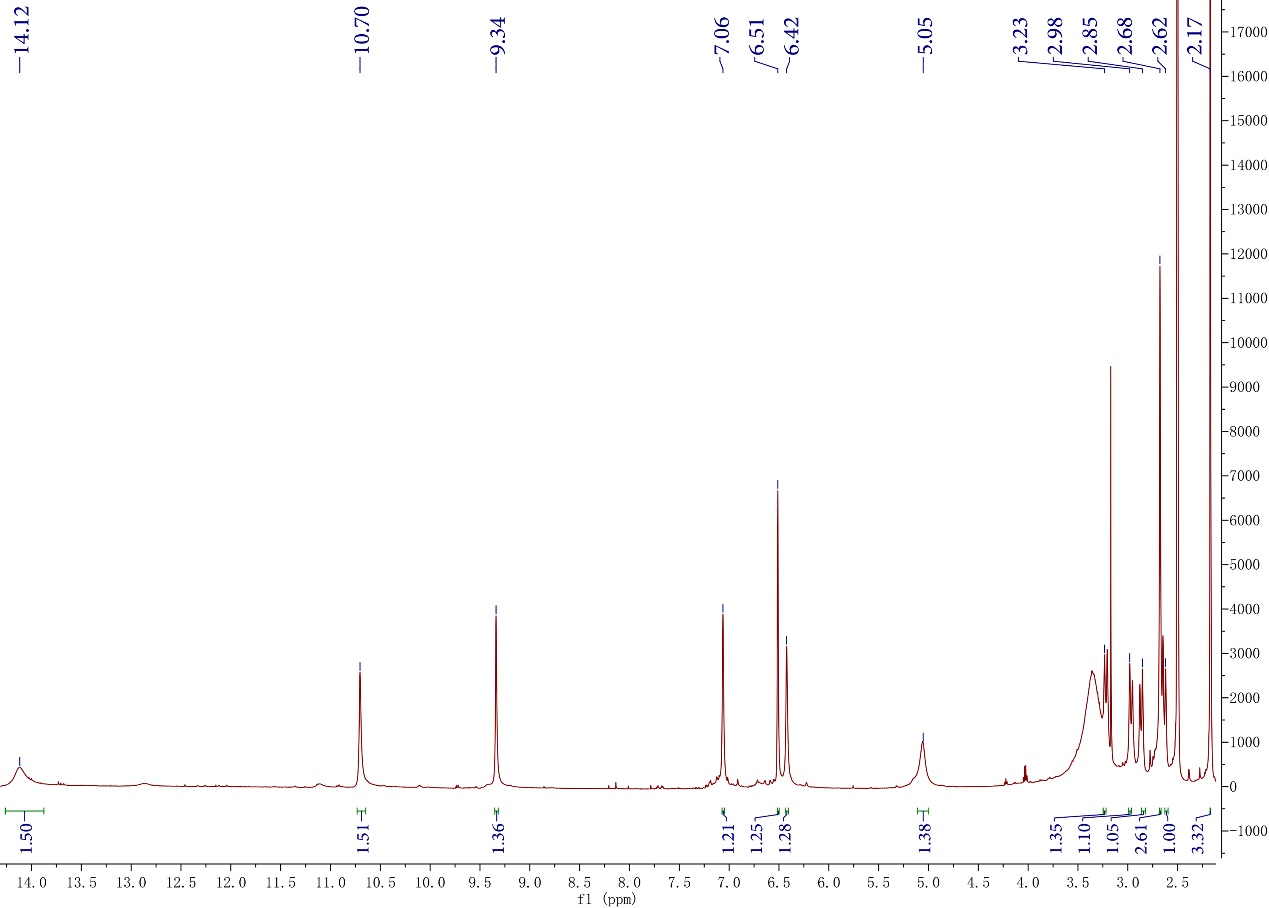


Supplementary Figure S13. ^1^H NMR spectrum (DMSO-*d*_6_, 600 MHz at 25℃) of lanthomicin A.


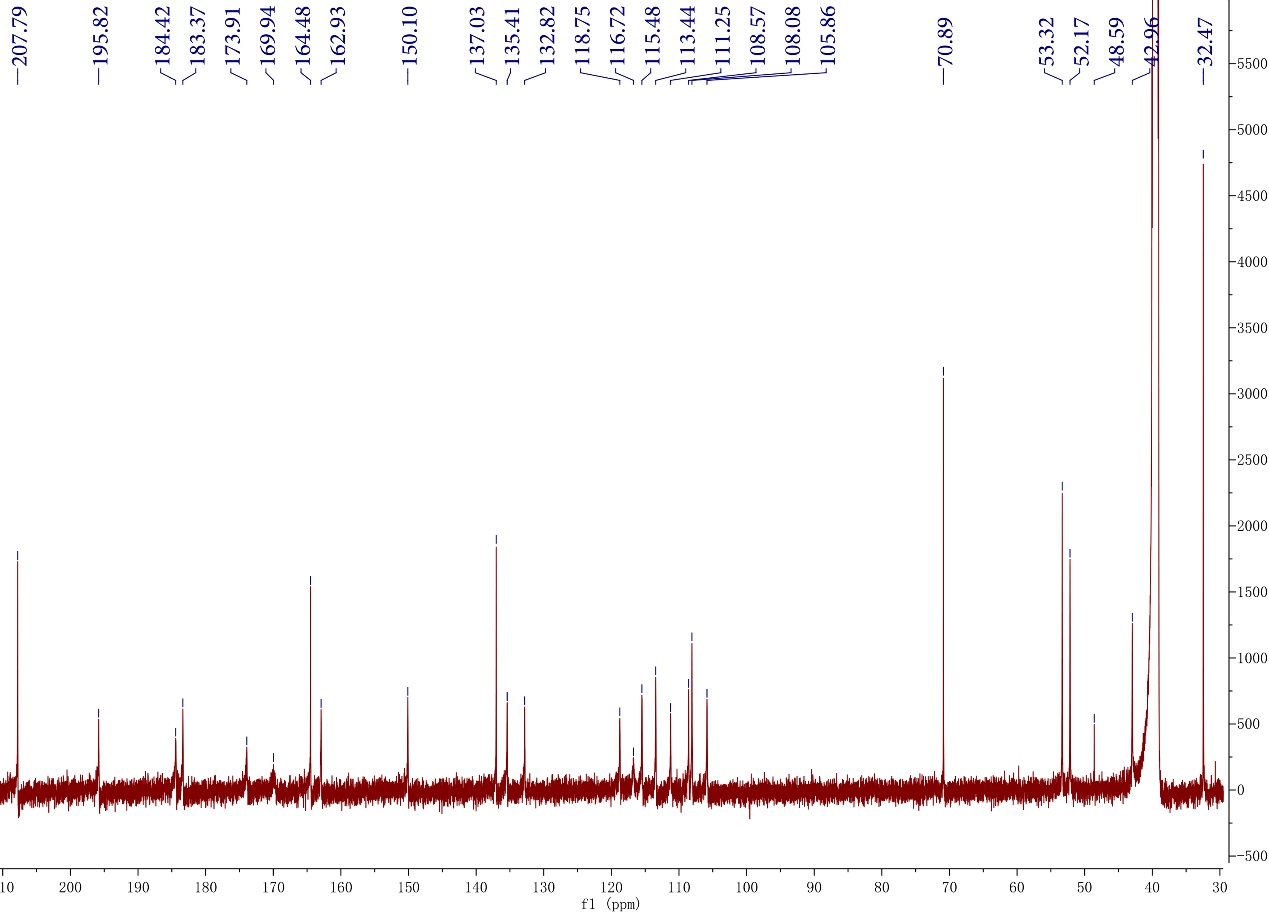


Supplementary Figure S14. ^13^C NMR spectrum (DMSO-*d*_6_, 125 MHz at 25℃) of lanthomicin A.


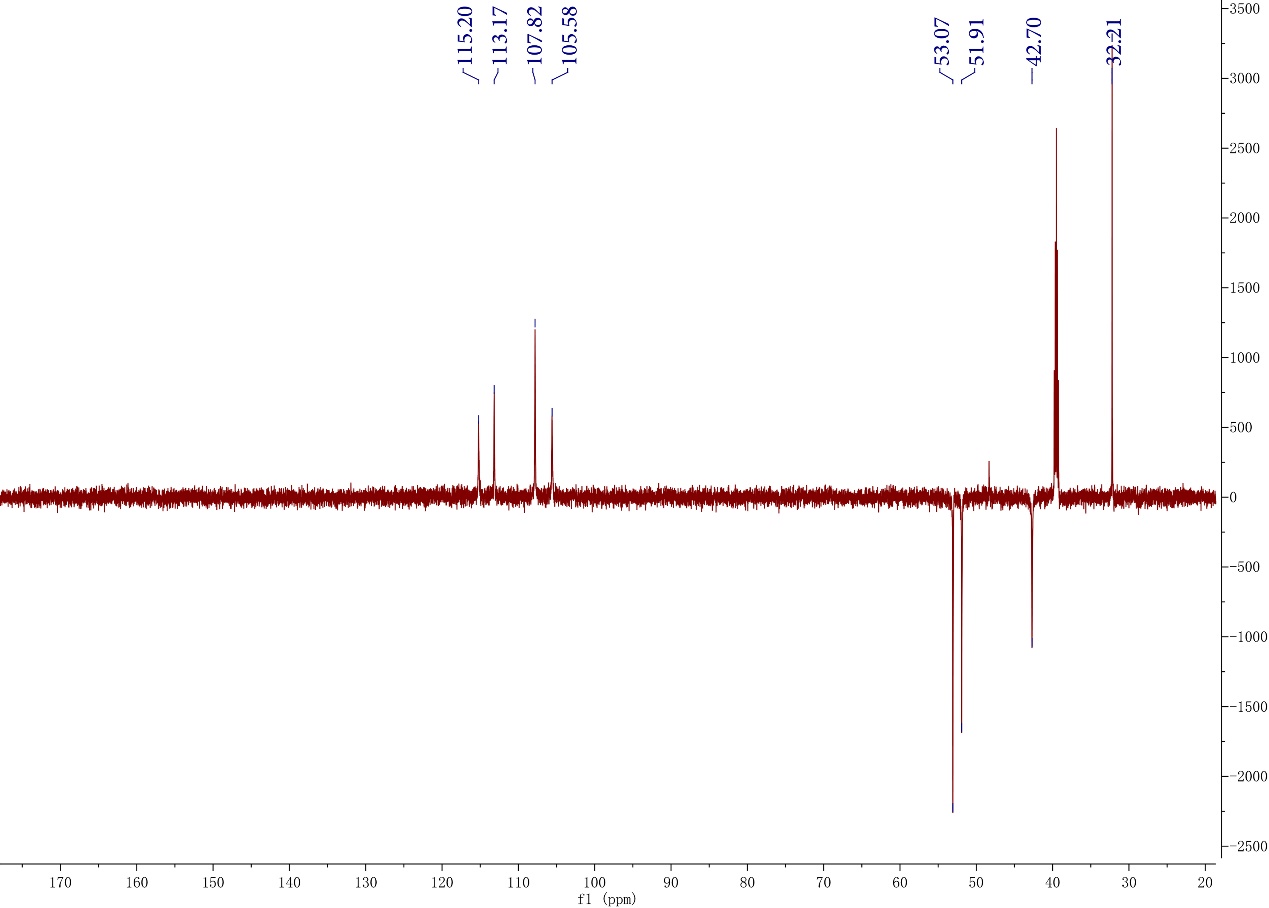


Supplementary Figure S15. DEPT 135 spectrum (DMSO-*d*_6_, 125 MHz at 25℃) of lanthomicin A.


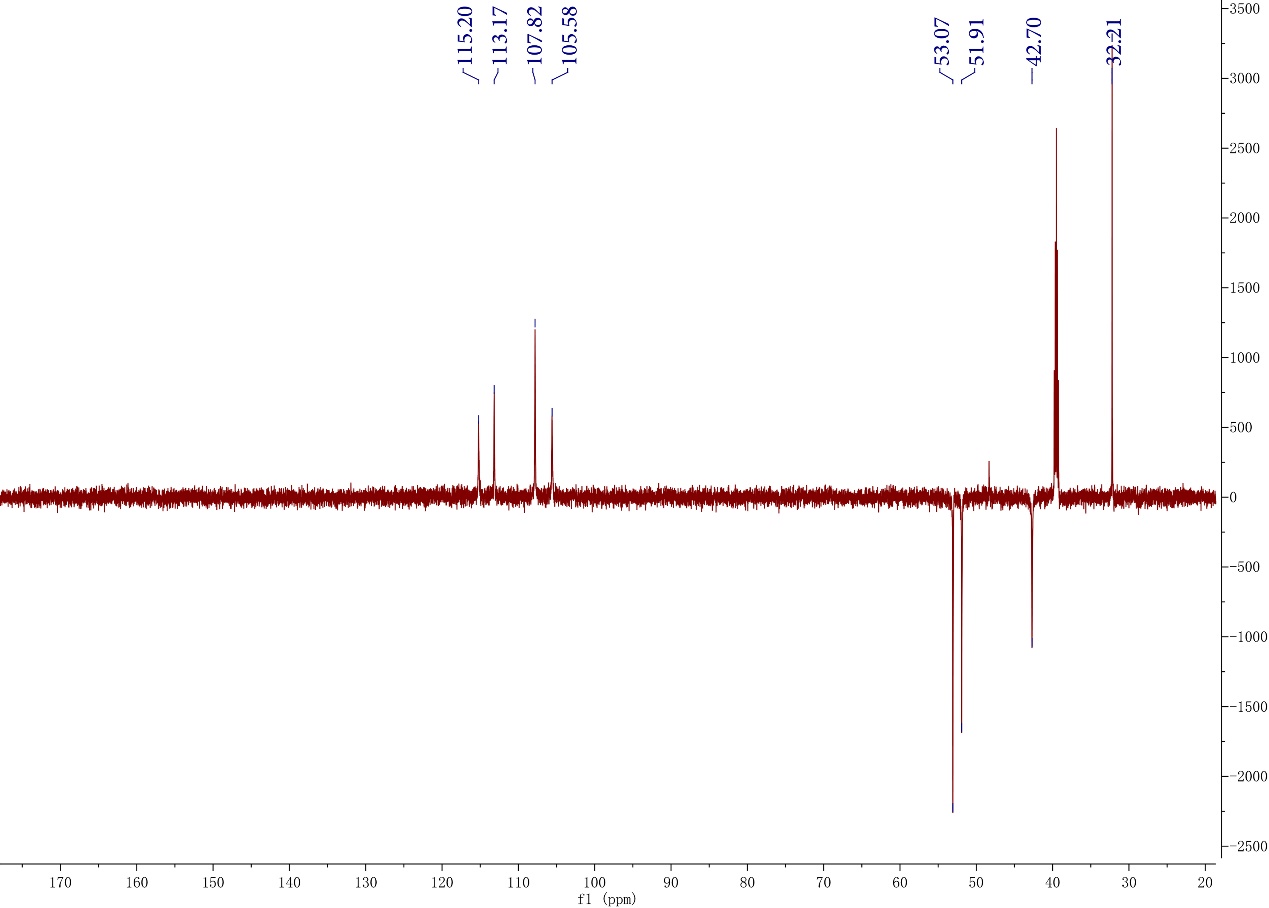


Supplementary Figure S16. COSY spectrum of lanthomicin A.


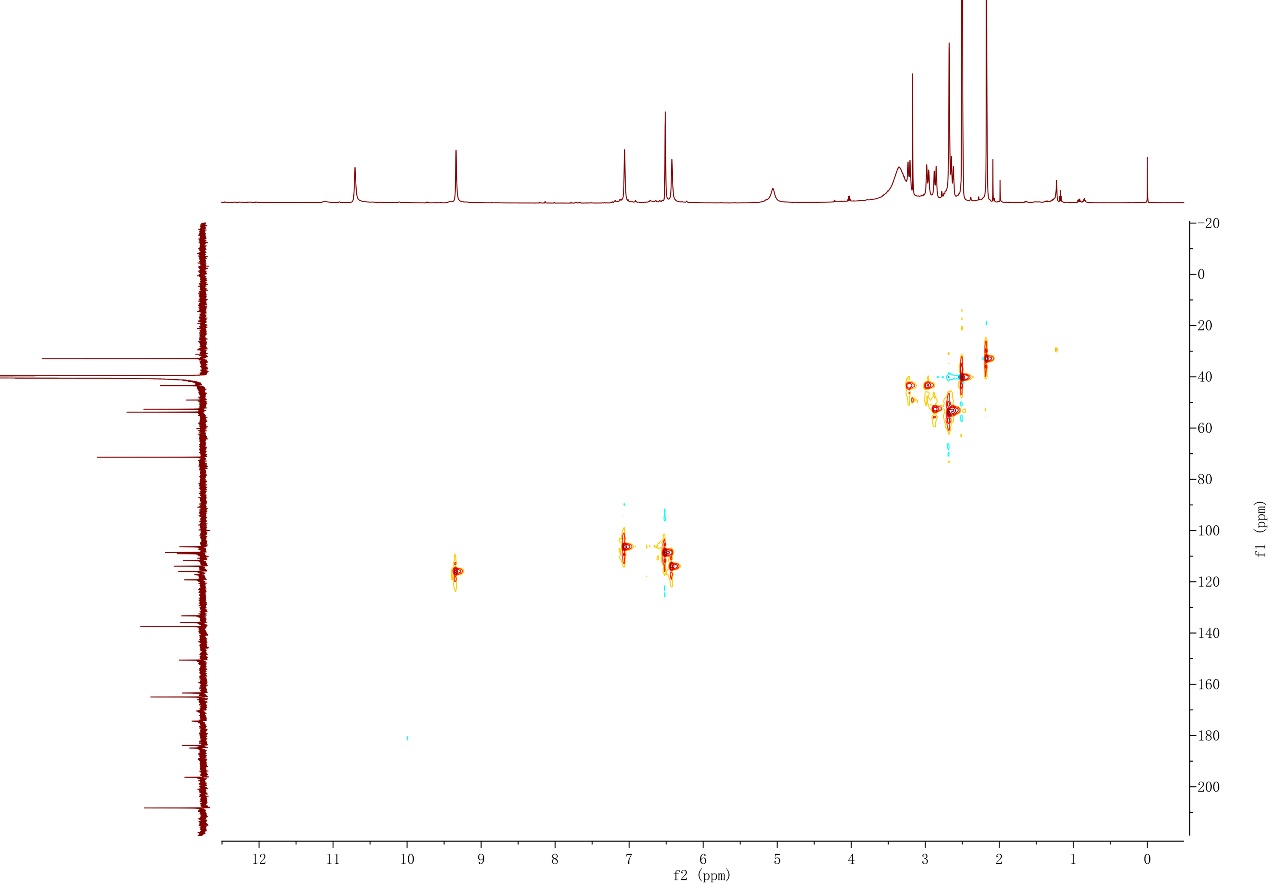


Supplementary Figure S17. HSQC spectrum of lanthomicin A.


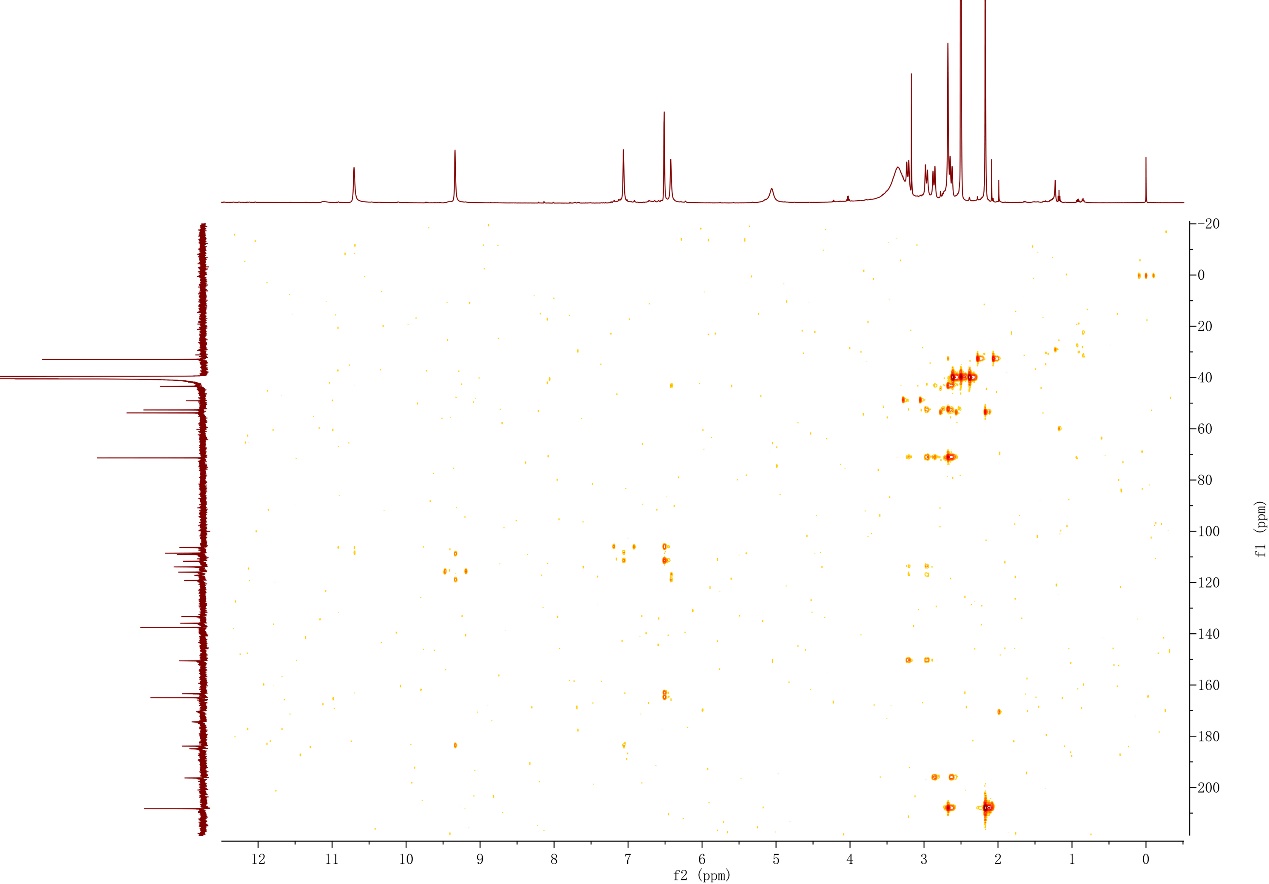


Supplementary Figure S18. HMBC spectrum of lanthomicin A.


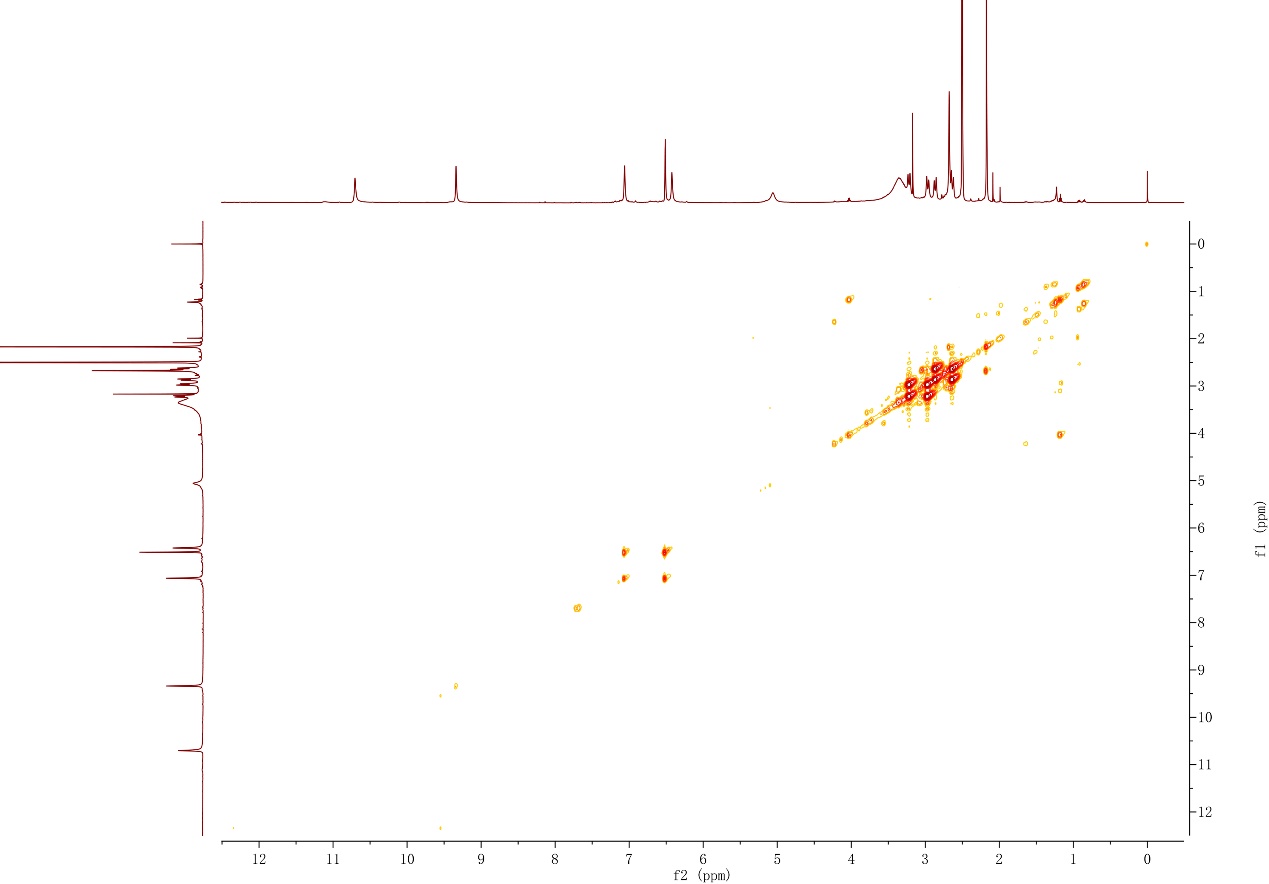


Supplementary Figure S19. NOESY spectrum of lanthomicin A.


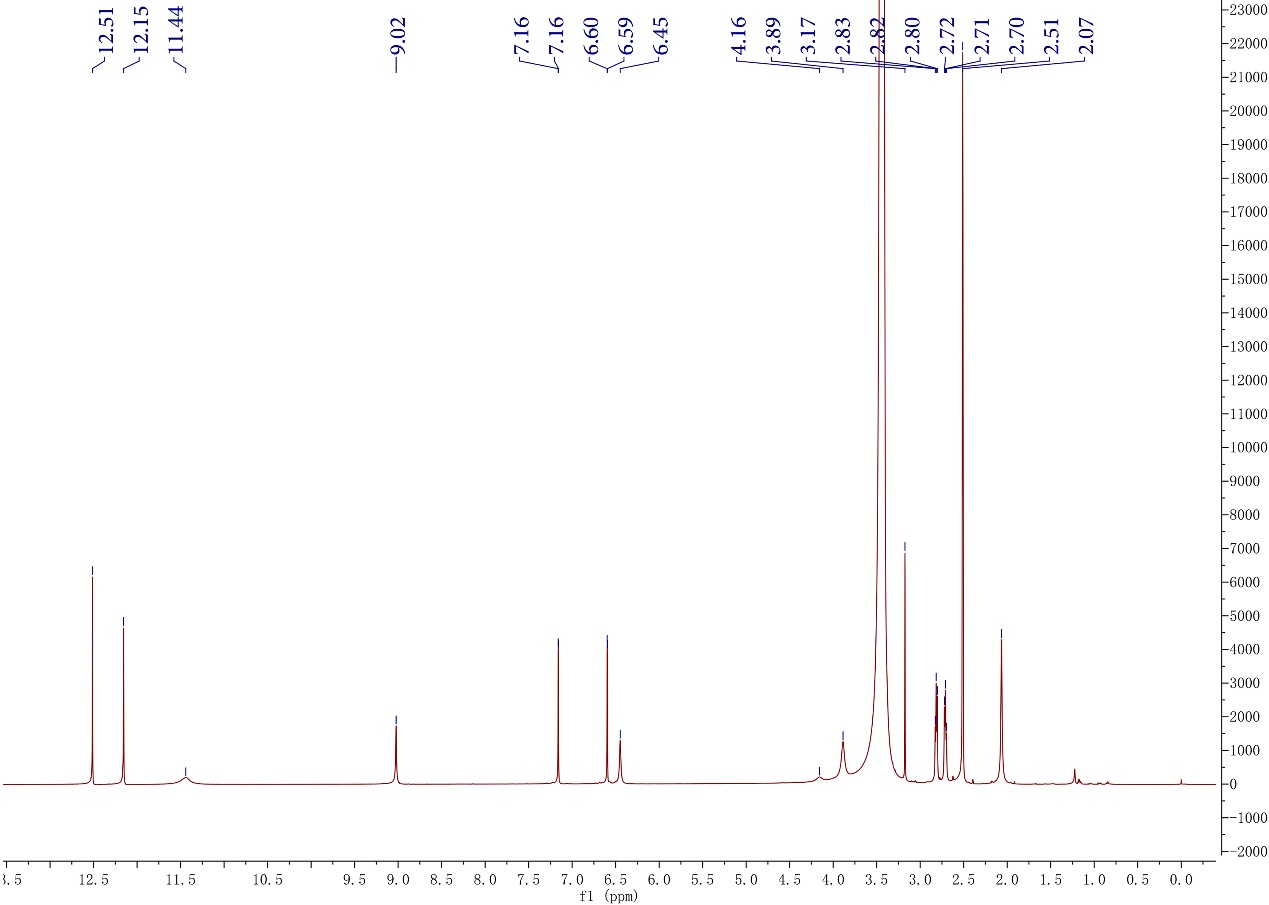


Supplementary Figure S20. ^1^H NMR spectrum (DMSO-*d*_6_, 600 MHz at 25℃) of lanthomicin B.


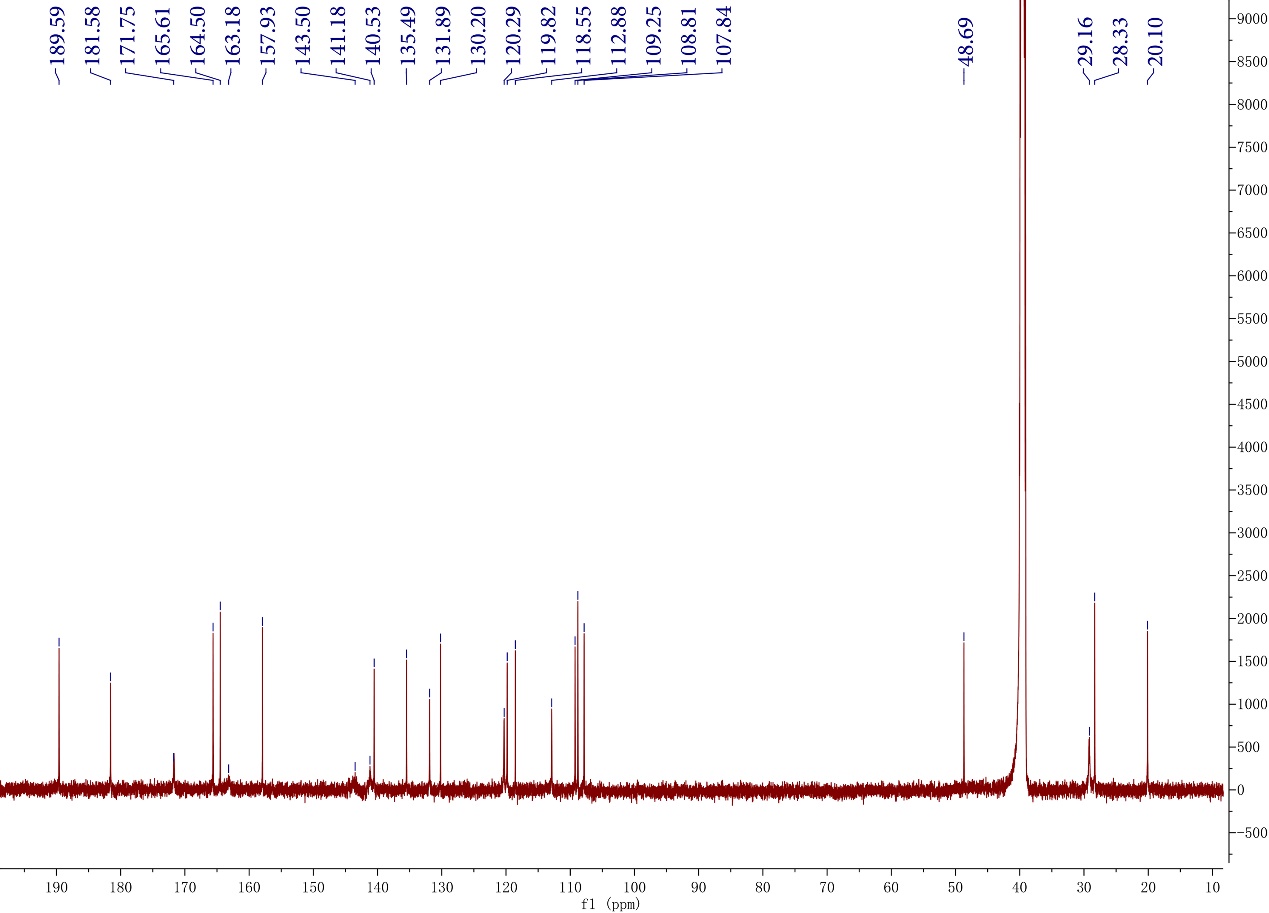


Supplementary Figure S21. ^13^C NMR spectrum (DMSO-*d*_6_, 125 MHz at 25℃) of lanthomicin B.


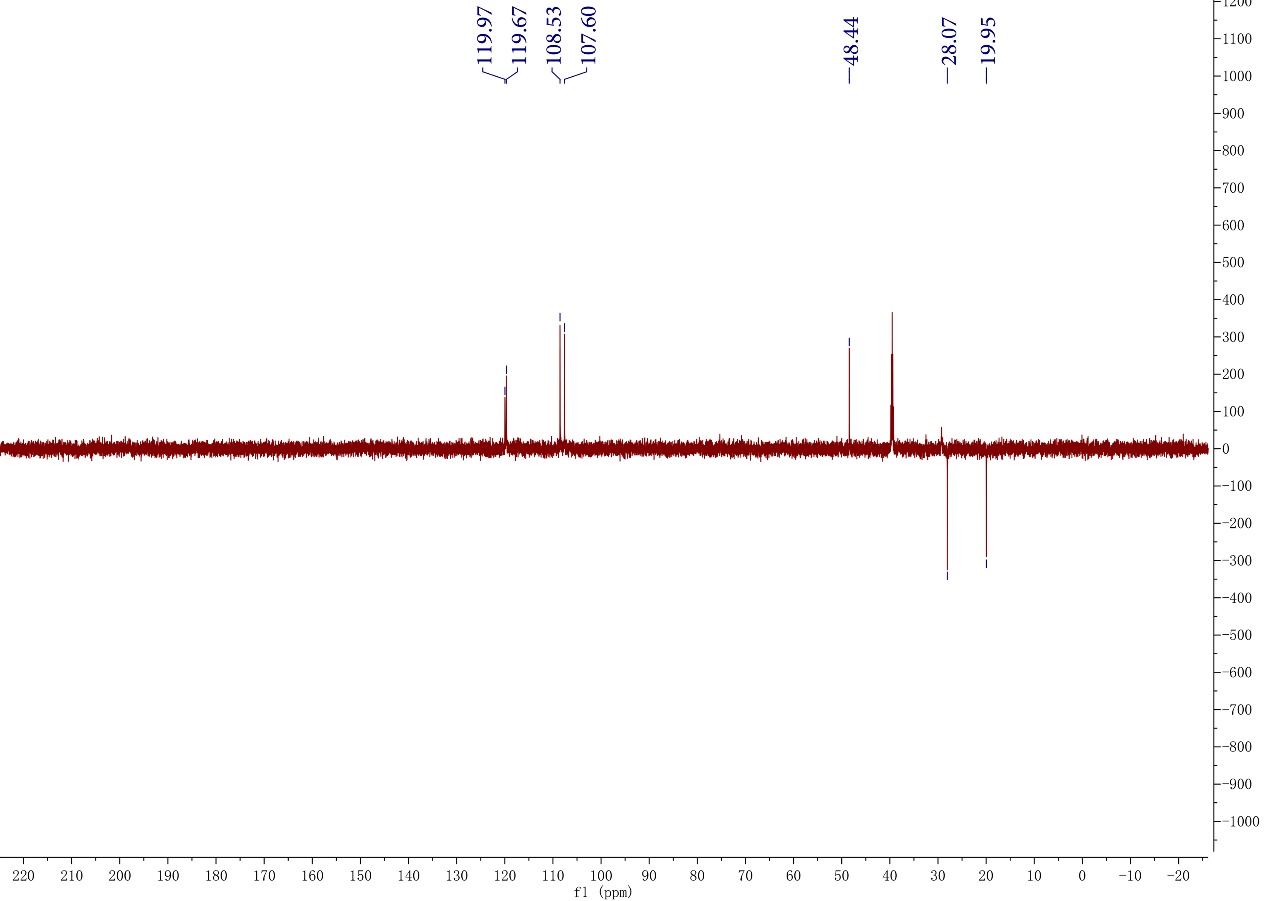


Supplementary Figure S22. DEPT 135 spectrum (DMSO-*d*_6_, 125 MHz at 25℃) of lanthomicin B.


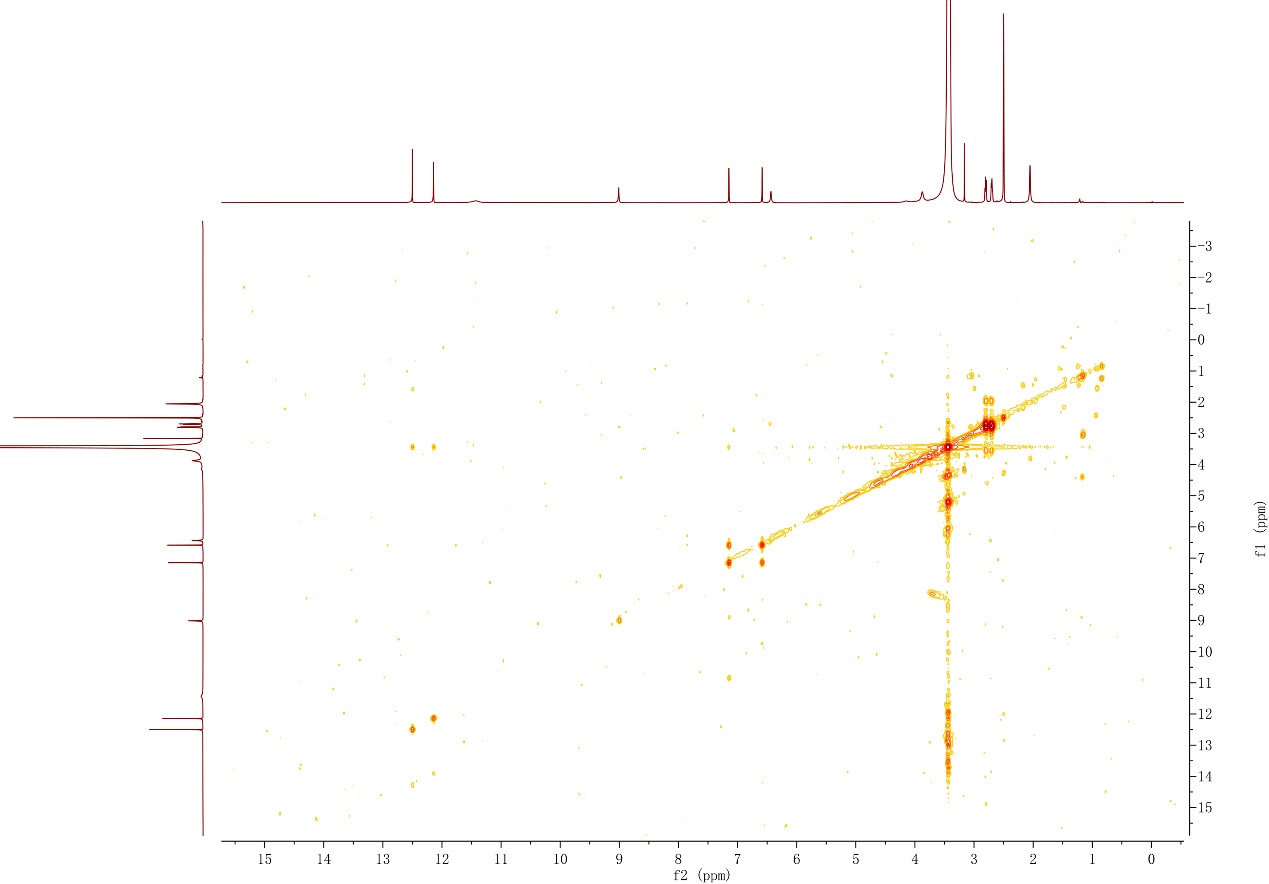


Supplementary Figure S23. COSY spectrum of lanthomicin B.


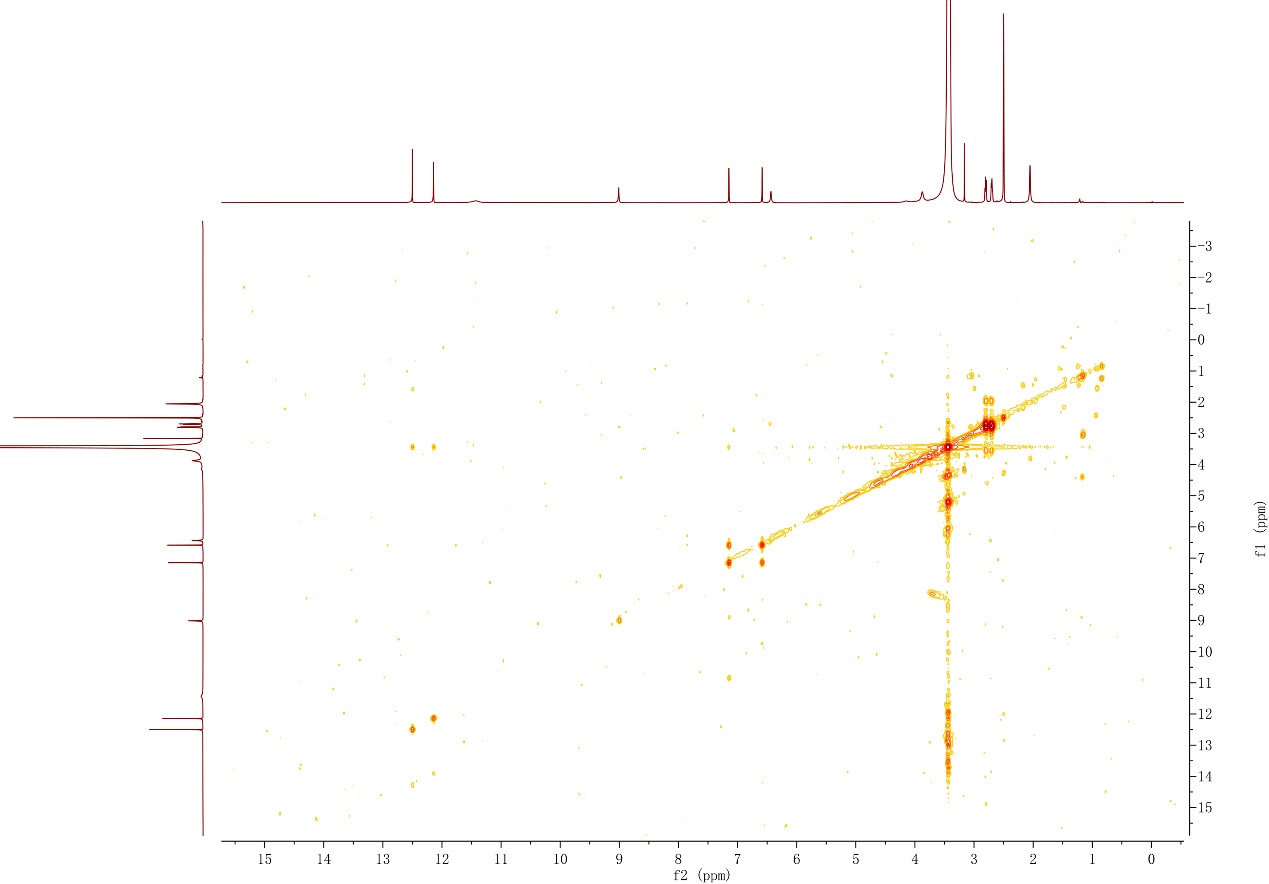


Supplementary Figure S24. HSQC spectrum of lanthomicin B.


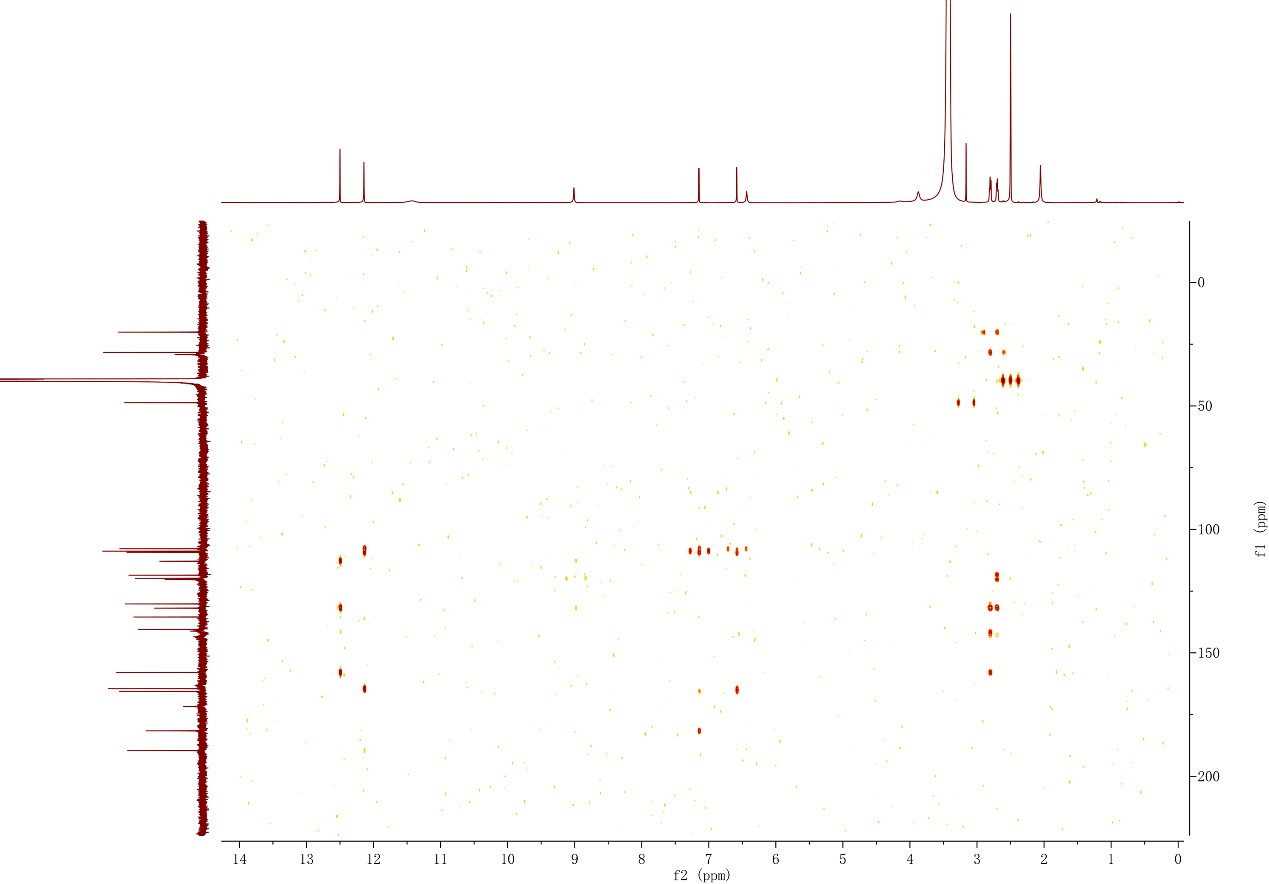


Supplementary Figure S25. HMBC spectrum of lanthomicin B.


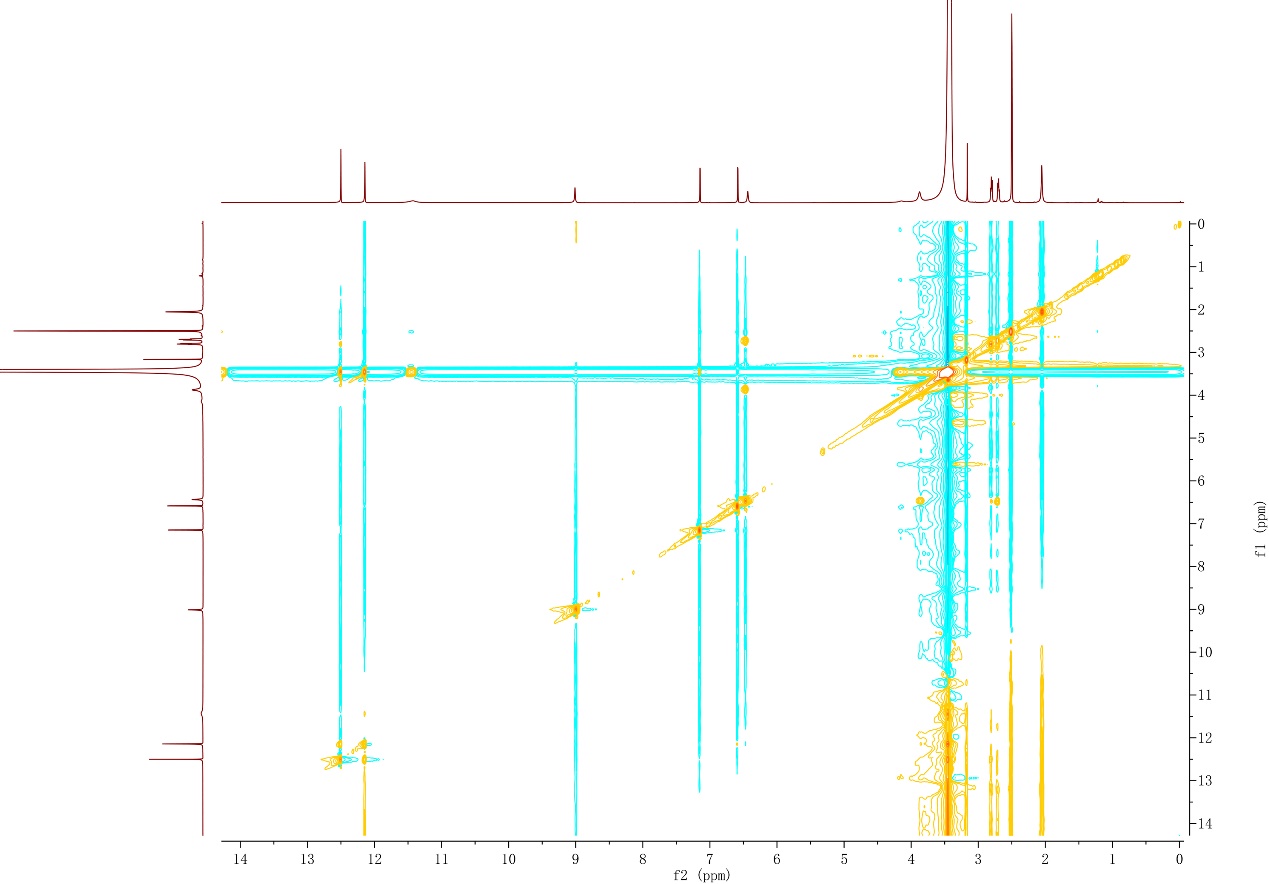


Supplementary Figure S26. NOESY spectrum of lanthomicin B.


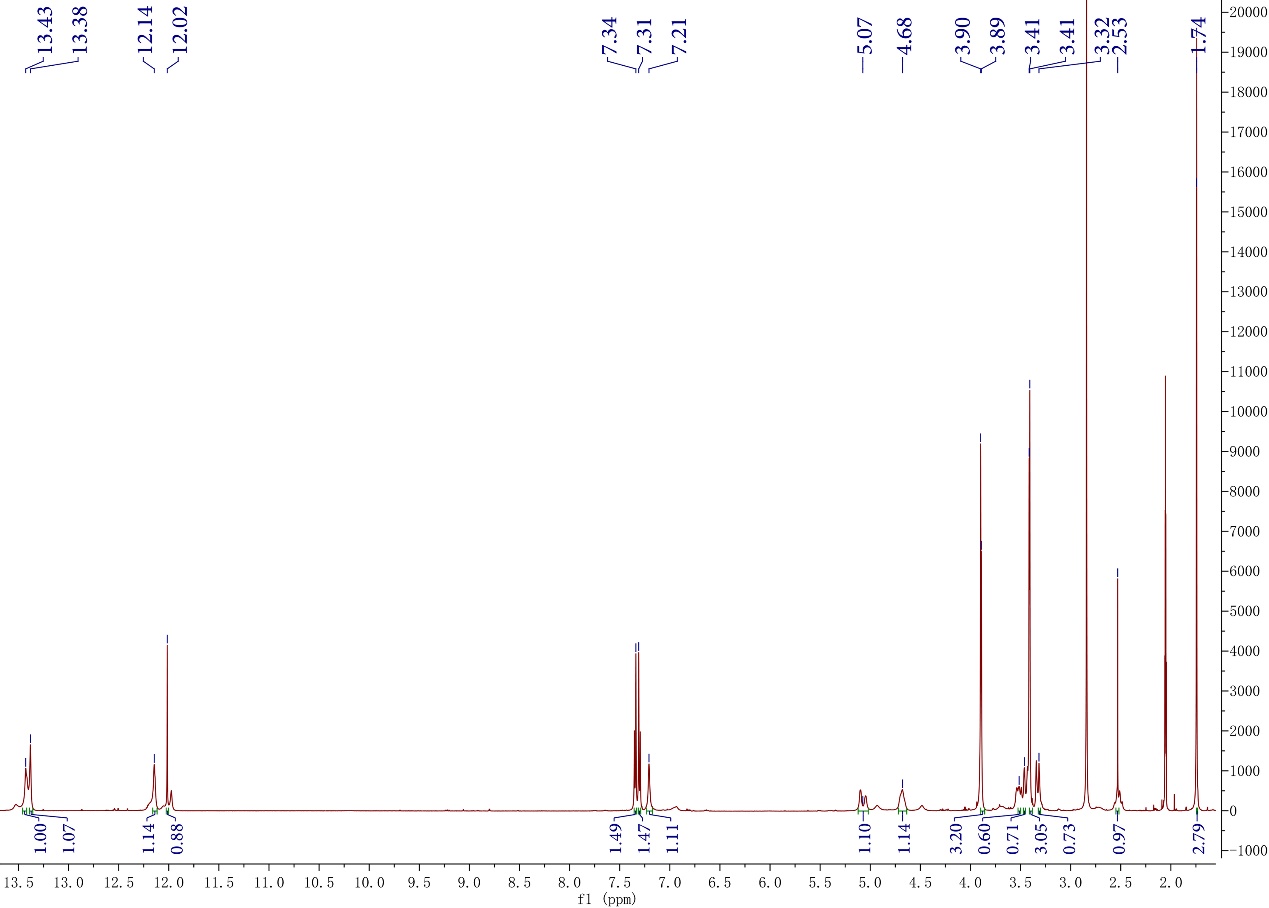


Supplementary Figure S27. ^1^H NMR spectrum (acetone-*d*_6_, 600 MHz at 25℃) of lanthomicin C.


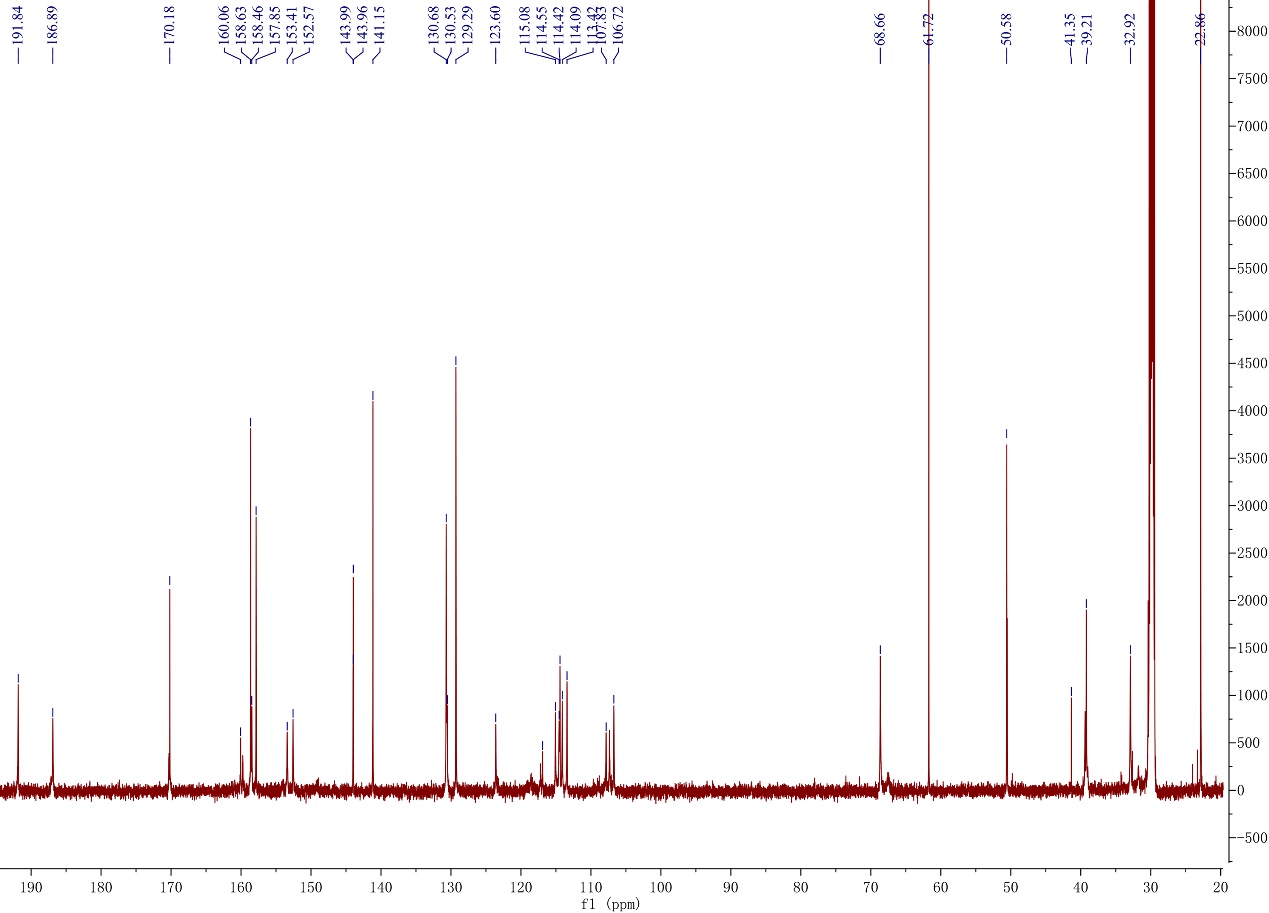


Supplementary Figure S28. ^13^C NMR spectrum (acetone -*d*_6_, 125 MHz at 25℃) of lanthomicin C.


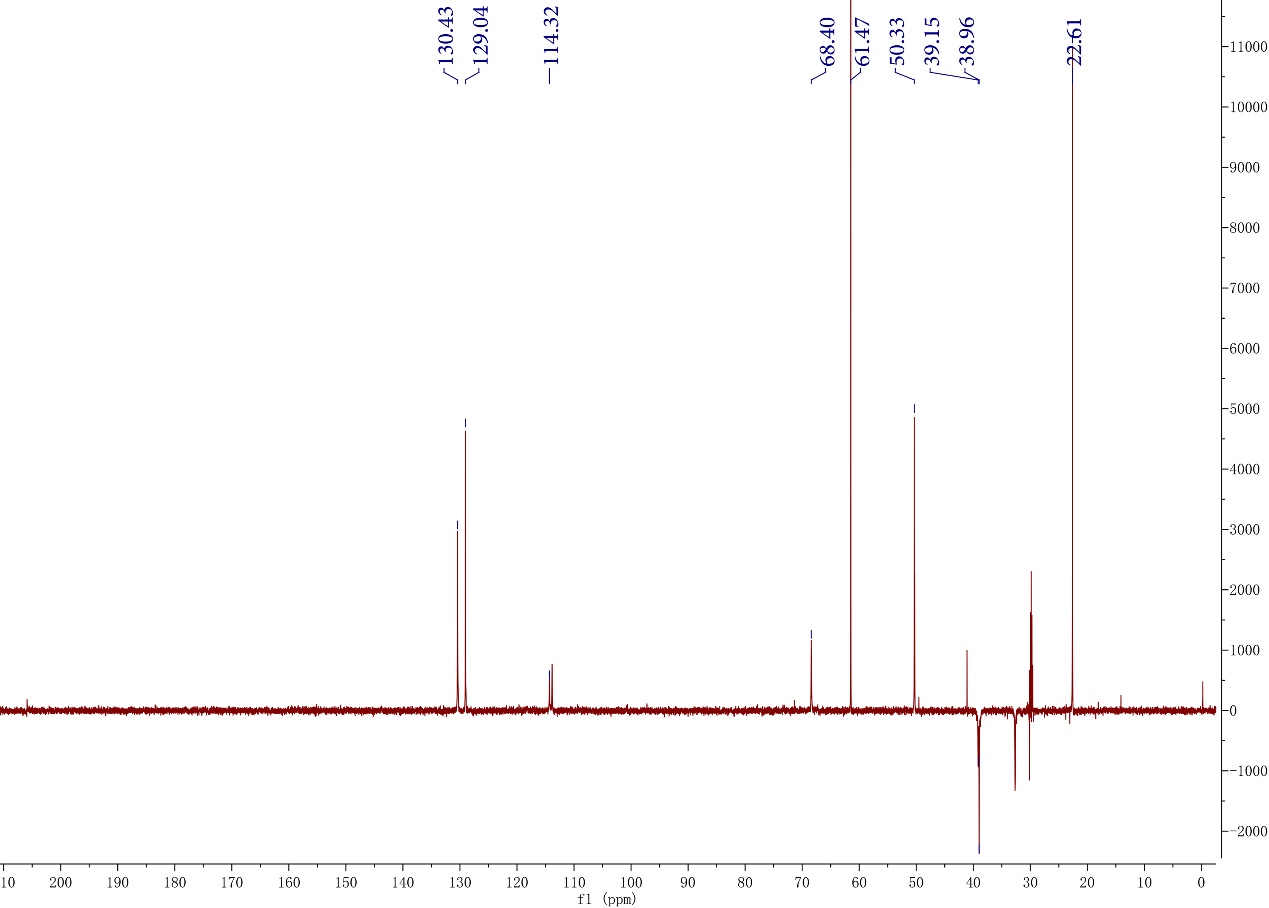


Supplementary Figure S29. DEPT 135 spectrum (acetone-*d*_6_, 125 MHz at 25℃) of lanthomicin C.


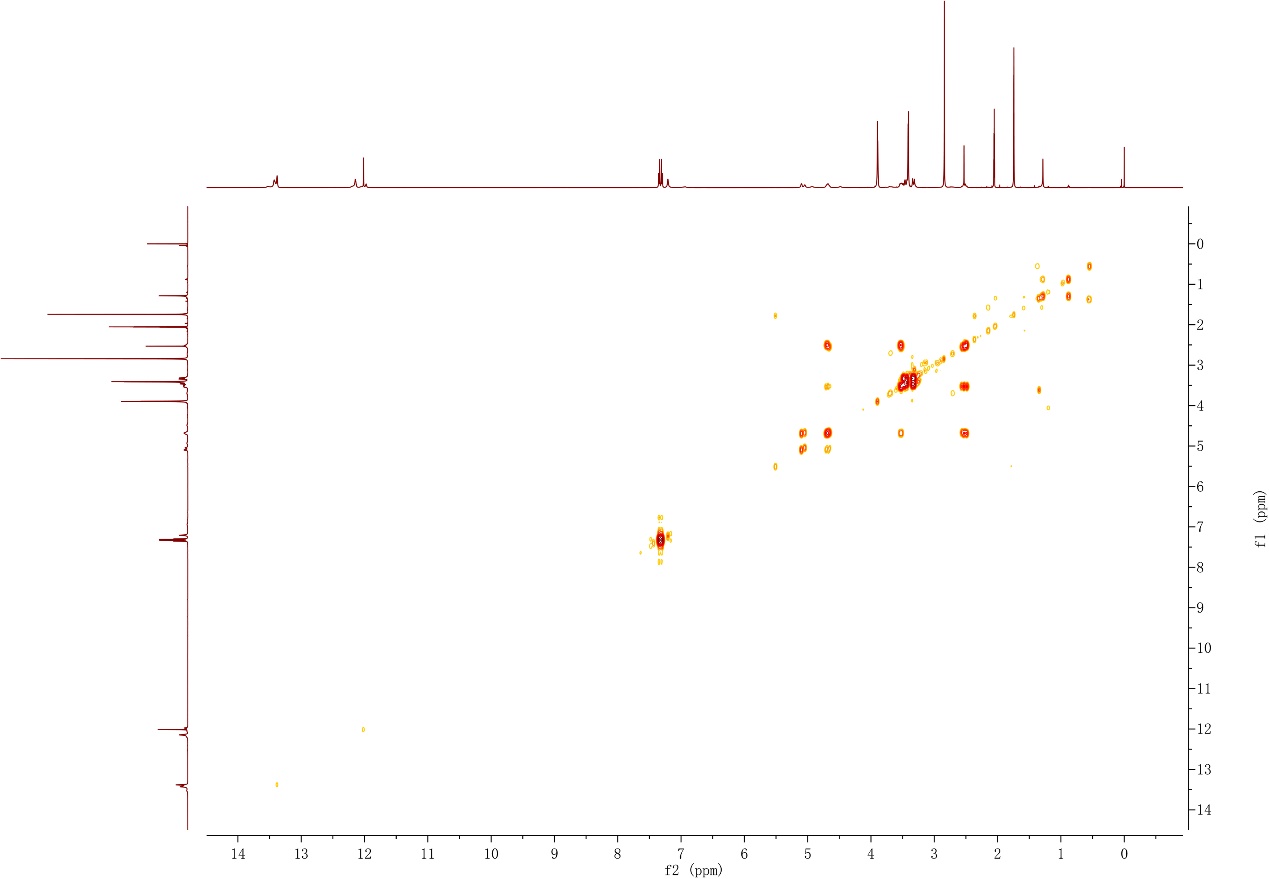


Supplementary Figure S30. COSY spectrum of lanthomicin C.


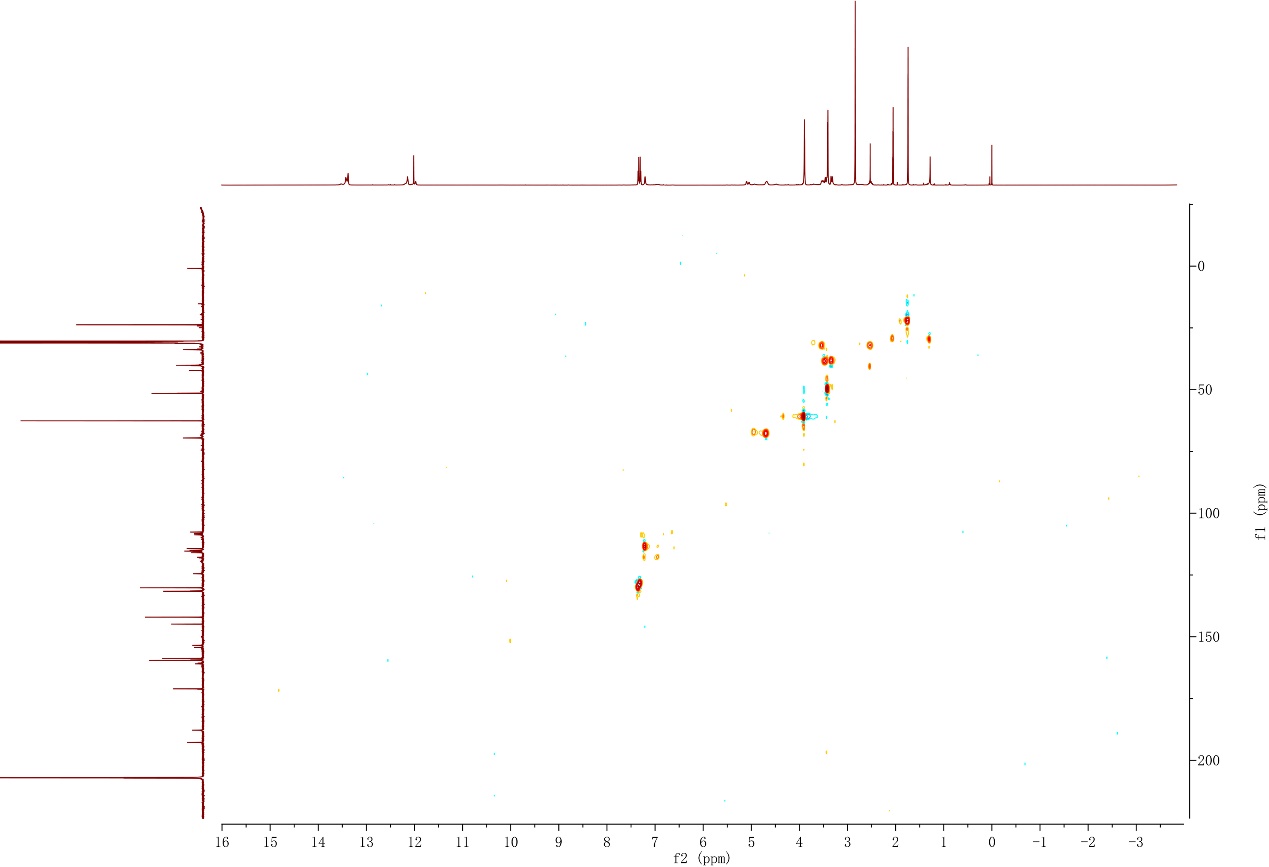


Supplementary Figure S31. HSQC spectrum of lanthomicin C.


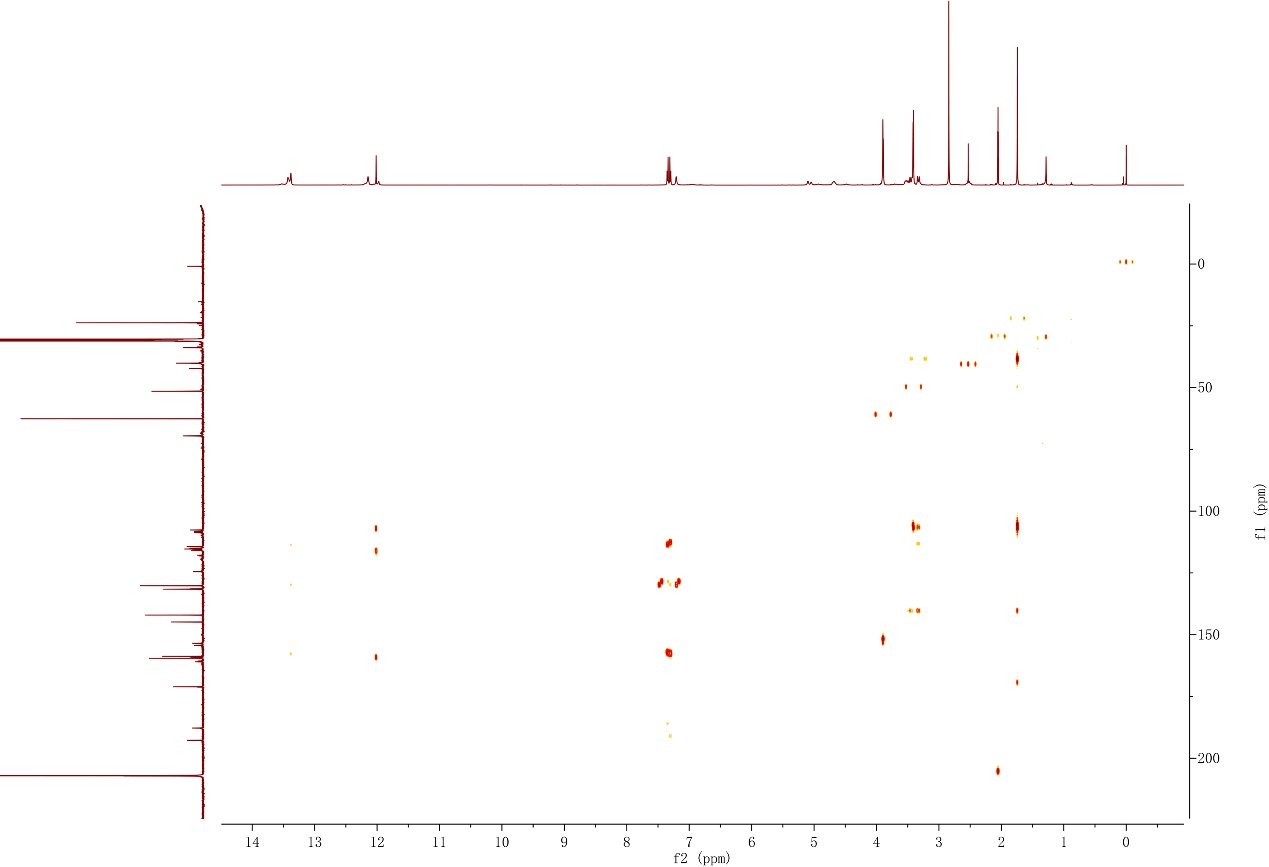


Supplementary Figure S32. HMBC spectrum of lanthomicin C.


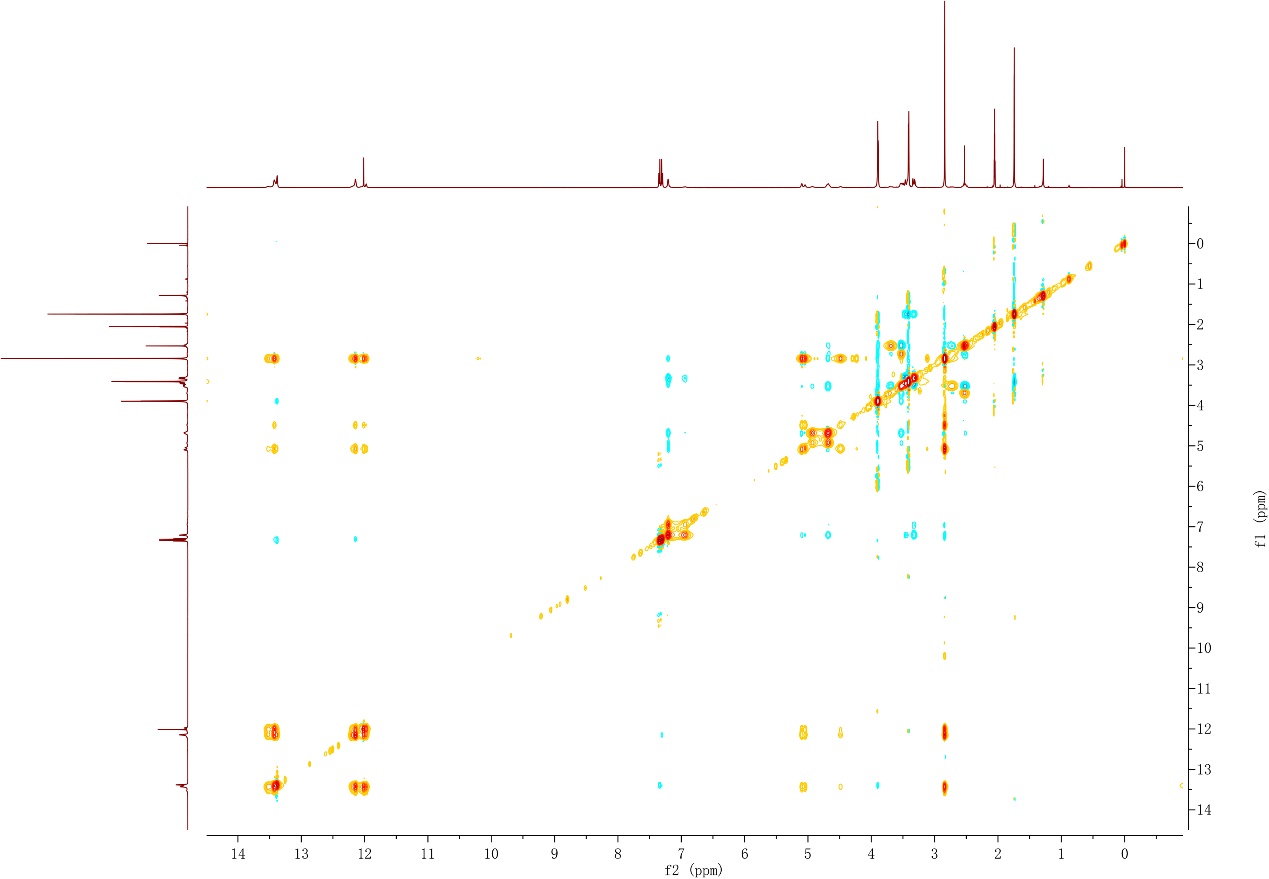


Supplementary Figure S33. NOESY spectrum of lanthomicin C.
