# Supplementary material for: Activation and Characterization of Lanthomicins A–C by Promoter Engineering in Streptomyces chattanoogensis L10
Source: Front Microbiol. 2022 May 10;13:902990. doi: 10.3389/fmicb.2022.902990 (PMC9127795; doi:10.3389/fmicb.2022.902990)
Supplement: Supplementary file 7 [file Data_Sheet_2.docx]

**Supplementary Methods**

**Plasmids construction**

**pXF01**: pSET152-spec-*kasO**-ltmR1. Primers ltmR1-F and ltmR1-R were used to clone the gene sequence of *ltmR1*, then the segment was linked to *Nde*I-digested pSET152-spec-*kasO** by recombination to gain pSET152-spec-*kasO**-ltmR1.

**pXF02**: pKC1139-spec-UHA-ltmR2-DHA. Primer pairs delltmR2-SF/SR and delltmR2-XF/XR were used to obtain the upstream and downstream homologous arms and cloned into the *Hind*III/*EcoR*V-digested pKC1139-spec by *in vitro* recombination to acquire pKC1139-spec-UHA-ltmR2-DHA.

**pXF03**: pSET152-spec-*kasO**-ltmF1D1D2ABCD3. Primer pairs cassatte-F1/R1 and cassatte-F2/R2 were used to amplify an upstream 2449bp fragment and a downstream 2388bp fragment of the *ltmF1D1D2ABCD3* multicistron with a 43bp overlap and then cloned into the *Nde*I-digested plasmid pSET152-spec-*kasO** by seamless cloning, affording an intact 7-gene open reading frame (ORF) under the control of constitutive *kasO** promoter.

**pXF04**: pKC1139-spec-UHA-*KmkasO**p-DHA. In order to substitute the native promoter upstream of *ltmF1*, primer pairs ltmF1-SF/SR, Km*kasO**p-F/*kasO**p-R2, and ltmF1-XF/XR were used to amplify a 1256bp upstream homologous arm, a *KmkasO**p cassette in which kanamycin resistence gene transcribed in an opposite direction, and a 1258bp downstream homologous arm, and the three segments were cloned into the *Hind*III/*EcoR*V-digested plasmid pKC1139-spec, resulting plasmid pKC1139-spec-UHA-*KmkasO**p-DHA.

**pXF05**: pKCCpf1(*tipA*p)-ltmAspacer. Primer pair ltmAspacer-F/R were used to amplify the ltmAspacer-crRNA-*kasO**p-*tipA*p cassatte using plasmid pKCCpf1(*tipA*p) as template, the cassatte targeting the functional domain of *ltmA* was then cloned into the *Spe*I/*Nde*I digested pKCCpf1(*tipA*p), affording plasmid pKCCpf1(*tipA*p)-ltmAspacer.

**pXF06**: pKCCpf1(*tipA*p)-ltmAspacer-HA. Primer pairs delltmA-SF/SR and delltmA-XF/XR were used to amplify the left and right arms of the core domain (aa 6 to 246), and then cloned into the *EcoR*V digested plasmid pKCCpf1(*tipA*p)-ltmAspacer to afford pKCCpf1(*tipA*p)-ltmAspacer-HA.

**pXF07**: pSET152-spec-*kasO**-ltmA. Primers ltmA-F and ltmA-R were for the amplification of *ltmA* gene segment, which was then inserted into *Nde*I-digested pSET152-spec-*kasO**, affording pSET152-spec-*kasO**-ltmA

***E. coli-Streptomyces* conjugation**

Plasmids were transferred to *E. coli* ET12567/pUZ8002 which was used as donor strain during conjugation via chemical transformation. Three single clones were picked and inoculated into a 250ml flask contain 50ml LB liquid medium with appropriate antibiotics. When the value of OD_600_ was about 0.5, the culture was harvested by centrifuging and the supernatant was discarded. The sediment was resuspended by adding 1ml of fresh LB medium and vortexed gently with a pipette. This process was repeated for three times to remove the residual antibiotics. A volume of 50μl spore suspension that was freshly prepared containing about 10^6^ viable spores was washed twice with 1ml 2×YT liquid medium and kept in 45℃ water for 10 min to germinate. Then both of the suspension were mixed together, centrifuged to discard most of supernatant, and the rest was used to resuspend the deposit and spread on MS plate. Nalidixic acid and spectinomycin were spread on the MS plate 18~20 hrs later and continued to culture.

**RNA extraction and quantitative RT-PCR experiment**

Mycelia of *S. chattanoogensis* L10 and its derivatives from YEME fermentation media were collected at 24h and frozen immediately for RNA extraction. The total RNA was prepared with sonication. Genomic DNA was removed with RNase-free DNase I (Takara). RNA concentration was determined using the Nanodrop Lite spectrophotometer (Thermo Scientific Co.). The cDNA was synthesized with MMLV reverse transcriptase according to the protocol of the manufacturer (Takara). qRT-PCR was performed using SYBR PremixEx Taq II (Takara). The sigma factor gene *hrdB* was used as an internal control for qRT-PCR assays in *S. chattanoogensis*. Each qRT-PCR was performed in triplicates. The fold changes of *ltm* gene expression levels were calculated by the comparative Ct method formulas according to the manufacturer’s protocol (Takara).
